# Effect of Sodium Bicarbonate on Indicators of Volume Retention in Metabolic Acidosis After Kidney Transplantation: A Post-Hoc Analysis

**DOI:** 10.1016/j.xkme.2026.101290

**Published:** 2026-02-11

**Authors:** Pierina S. Staub, David Kronthaler, Anna Wiegand, Suzan Dahdal, Daniel Sidler, Spyridon Arampatzis, Karine Hadaya, Harald Seeger, Rudolf P. Wüthrich, Ulrike Held, Carsten A. Wagner, Nilufar Mohebbi, Alexander Ritter

**Affiliations:** 1 Institute of Physiology, University of Zurich, Zurich, Switzerland and Zurich Kidney Center; 2 Department of Biostatistics at Epidemiology, Biostatistics and Prevention Institute, University of Zurich, Zurich, Switzerland; 3 Division of Nephrology, University Hospital Zurich, Zurich, Switzerland; 4 Division of Nephrology and Hypertension, Inselspital, Bern, Switzerland; 5 Clinique des Grangettes, Hirslanden, Geneva, Switzerland; 6 Institute for Nephrology and Dialysis, Cantonal Hospital Baden, Baden, Switzerland; 7 Division of Nephrology and Transplantation Medicine, HOCH, Cantonal Hospital St.Gallen, St. Gallen, Switzerland

**Keywords:** sodium bicarbonate, metabolic acidosis, kidney transplantation, cardiovascular, blood pressure, hypertension, body weight, N-terminal prohormone of brain natriuretic peptide (NT-proBNP), renin, aldosterone, potassium, antihypertensive medication, diuretics, hospitalization

## Abstract

**Rationale & Objective:**

Metabolic acidosis is a frequent complication after kidney transplantation, commonly treated with sodium bicarbonate. However, concomitant sodium loading raises concerns about potential volume retention. This study aims to assess its impact on clinical and biochemical markers of volume retention, as its effects post-transplant remain insufficiently studied.

**Study Design:**

Post-hoc analysis of a randomized, single-blinded, placebo-controlled multicenter trial (Preserve-Transplant Study).

**Setting & Participants:**

Total of 240 kidney transplant recipients with metabolic acidosis were randomized 1:1 across 3 University hospitals in Switzerland.

**Intervention:**

1.5-4.5 g/day sodium bicarbonate or placebo for 24 months.

**Outcomes:**

Primary outcomes included body weight, N-terminal prohormone of brain natriuretic peptide, plasma renin, plasma aldosterone, aldosterone-to-renin ratio (the latter 4 log-transformed), nocturnal systolic blood pressure dipping, and antihypertensive agent use. Secondary outcomes included the occurrence of hypertension, hospitalization due to uncontrolled hypertension or volume overload, and sodium and potassium balance over time.

**Analytical Approach:**

Descriptive statistics and mixed-effects regression models.

**Results:**

There was weak evidence for treatment effects in the sodium bicarbonate group compared with the placebo group regarding body weight (between-group difference [BGD]: 1.23 kg, 95% CI: −0.18 to 2.63; *P* = 0.09), no evidence for N-terminal prohormone of brain natriuretic peptide (BGD: −0.03, 95% CI: −0.21 to 0.16; *P* = 0.78), plasma renin (BGD: −0.12, 95% CI: −0.34 to 0.09; *P* = 0.26), and aldosterone-to-renin ratio (BGD: −0.05, 95% CI: −0.28 to 0.18; *P* = 0.69), whereas there was substantial evidence for a treatment effect on plasma aldosterone (BGD: −0.17, 95% CI: −0.3 to −0.05; *P* = 0.007), the latter 4 log-transformed. There was weak evidence for an increased chance of nocturnal systolic blood pressure dipping (OR: 2.28; 95% CI: 0.88 to 5.87; *P* = 0.09). The use of antihypertensives and diuretics remained similar in both groups. Weak evidence for higher serum sodium (BGD: 0.51 mEq/L, 95% CI: 0.08 to 0.94; *P* = 0.02) and strong evidence for higher log-transformed 24 hour-sodium excretion (BGD: 0.16, 95% CI: 0.07 to 0.26; *P* ≤ 0.001) in the sodium bicarbonate group were identified. There was no or weak evidence for treatment effects on other secondary outcomes. Subgroup analyses showed no major differential effects.

**Limitations:**

Post-hoc analysis, mainly Caucasian study population.

**Conclusions:**

Our findings did not provide clear evidence for unfavorable effects of sodium bicarbonate on volume retention. Further research is required to clarify risks.

**Trial Registration:**

ClinicalTrials.gov (ID: NCT03102996).

## Plain-Language Summary

Many kidney transplant recipients develop metabolic acidosis because the transplanted kidney may not fully restore acid–base balance. Sodium bicarbonate is commonly used to correct this, but its associated sodium load raises concerns about fluid retention. This study compared long-term sodium bicarbonate use with placebo and evaluated signs of volume overload, including changes in body weight, blood markers linked to fluid regulation, blood pressure patterns, and the use of blood pressure medications. Moreover, hospitalizations related to uncontrolled blood pressure or fluid overload were studied. Overall, the results showed no clear evidence of harmful effects. Further research should confirm these findings.

Alkali treatment with sodium bicarbonate (SB) is a standard therapy for chronic metabolic acidosis (MA), a common complication caused by acid retention in advanced chronic kidney disease (CKD). Its prevalence increases with declining glomerular filtration rate (GFR) and is associated with increased morbidity and mortality.^1^, ^2^, ^3^ In kidney transplant recipients (KTRs), MA is even more prevalent (12%-58%)^4^ and occurs at higher GFR levels, likely due to additional pathophysiological mechanisms.^1^ In KTRs, MA is associated with low bone mineral density, cardiovascular events, graft loss, and increased overall mortality.^5^, ^6^, ^7^ In contrast to nontransplant CKD, a beneficial effect of SB treatment on GFR decline has not yet been demonstrated in KTRs.^8^, ^9^, ^10^

Despite its widespread use, the sodium load accompanying SB administration may cause volume retention, raising safety concerns, particularly without concomitant dietary sodium chloride (NaCl) restriction.^11^^,^^12^ Salt intake is a known cardiovascular risk factor, especially for hypertension.^13^ Patients with CKD often exhibit sodium-sensitive comorbidities such as hypertension and heart failure.^14^^,^^15^ Sodium bicarbonate treatment may increase the risk of congestive heart failure^16^^,^^17^ and attenuate cardiovascular protective effects of renin-angiotensin-aldosterone system (RAAS)-inhibitors.^18^ However, recent data from nontransplant patients with CKD are controversial. Several studies reported no significant change in body weight (BW),^8^ blood pressure (BP), or antihypertensive use with SB-treatment.^19^^,^^20^ Others described significantly higher systolic blood pressure (SBP),^8^ a significant increase in diastolic blood pressure, higher incidence of edema^21^ or increased use of diuretics^22^ in SB-treated patients.

In KTRs, these adverse effects of SB remain largely unexplored. Besides the primary analysis of the Preserve-Transplant Study,^10^ which found no safety signals and differences in BP, only one small randomized, placebo-controlled pilot study examined these aspects of SB treatment, and found no difference in BP or BW.^23^ Considering the high prevalence of MA in KTRs,^4^ these safety aspects of SB treatment in this often fragile population deserve further study.

The present study aimed to clarify the safety profile of SB in acidotic KTRs by assessing its effects on parameters indicative of volume retention, filling a critical gap in our understanding of this therapeutic approach.

## Methods

### Study Design and Participants

This study is a secondary analysis of the original Preserve-Transplant Study,^10^ a prospective, multicenter, randomized, single-blinded, placebo-controlled, and investigator-initiated trial primarily designed to assess the effect of oral SB on allograft function in KTRs with MA over 2 years. The trial results and protocol have been published.^10^^,^^24^ It was conducted in 3 Swiss transplant centers (Bern, Geneva, and Zurich). Patients were randomized 1:1 to receive either SB or placebo. Total of 240 KTRs were included in the modified intention-to-treat population (ITT) after secondary exclusion of 2 patients due to protocol violation or withdrawal of informed consent.^10^ Main eligibility criteria comprised age ≥18 years, ≥12 months after kidney transplantation, stable graft function (change in serum creatinine <15% within 6 months before inclusion), estimated GFR (eGFR) between 15-89 mL/min/1.73m^2^ and ≥1 serum bicarbonate measurement ≤22 mEq/L within the last 6 months. Uncontrolled hypertension or use of >4 antihypertensive drugs, uncontrolled heart failure, serum potassium <3.0 mEq/L, serum sodium >150 mEq/L, recent use of alkali within 4 weeks, or use of medication influencing MA led to exclusion. In this post-hoc analysis, all patients of the modified ITT were included. Ethical approval was obtained from the corresponding Cantonal ethics committees (Registration number: 2016–02012). The study was conducted according to the WMA Declaration of Helsinki. Patients gave written informed consent.

### Procedures

Patients initially received either oral SB or placebo with a dose of 1,500 mg (BW <70 kg) or 3,000 mg (BW ≥70 kg) per day. At titration visit (after 2 weeks) the daily dosage was raised by 1,500 mg to 3,000 mg or 4,500 mg if serum bicarbonate was ≤22 mEq/L. Each verum capsule (500 mg) contained 137 mg sodium, resulting in an additional daily sodium load of 411-1,233 mg. Study drug capsules (SB or placebo) with identical appearance ensured masking. Patients received no dietary restrictions. BW, office blood pressure (OBP), spot urine, and standard venous blood parameters (sodium and potassium) were assessed every 3 months. Concomitant medication was documented at each visit. At baseline, 12 and 24 months, 24-hour urine collections, ambulatory blood pressure monitoring (ABPM), serum N-terminal pro-brain natriuretic peptide (NT-proBNP), as well as plasma renin and aldosterone levels were obtained and measured under standardized conditions (including a 30-minute rest period before blood draw). In Bern and Geneva, renin and aldosterone samples were obtained in an ethylenediaminetetraacetic acid-plasma tube, centrifuged at room temperature, stored between −20 to −80 °C, and analyzed at a central laboratory (University Hospital Zurich) within 4 weeks. In Zurich, samples were analyzed in the central laboratory immediately after blood draw. Only ABPM with ≥70% successful BP measurements were included in the analysis.^25^ Hospitalizations and use of medications were recorded. Data was documented in a web-based secured data management system (SecuTrial).

### Outcomes

The primary outcomes of interest included BW, NT-proBNP, plasma renin, plasma aldosterone, and aldosterone-to-renin ratio up to 2 years of follow-up. Furthermore, the percentage of patients with ≥10% nocturnal systolic ABPM dipping and the percentage of patients with a change in the number of antihypertensive agents (including diuretics) and diuretics (separately) from baseline to 12 and 24 months were assessed. The number and dosage of antihypertensives (including diuretics) and diuretics (separately) according to substance class at 12 and 24 months compared to baseline were assessed. As secondary outcomes, mean intake of study medication in the SB group, serum sodium, serum potassium, and sodium and potassium excretion (24 hour-urine and spot urine as sodium/creatinine and potassium/creatinine ratio) were evaluated. Additionally, hypertension in OBP (≥140/90 mm Hg,^25^ mean of second and third measurement), hypertension in overall 24 hour-ABPM (≥130/80 mm Hg)^25^ and hospitalization due to uncontrolled hypertension or volume overload were assessed. Observed OBP and ABPM values have been published in detail in the supplements of the primary study.^10^

### Subgroup Analyses

Subgroup analyses were performed for primary outcomes, stratified by number of antihypertensive drugs including diuretics (1, 2, 3, and ≥4), eGFR range (15-<30 mL/min/1.73m^2^, 30-<45 mL/min/1.73m^2^, 45-<60 mL/min/1.73m^2^, and ≥60 mL/min/1.73m^2^), serum bicarbonate levels (≤18 mEq/L, 18-<20 mEq/l, 20-<22 mEq/L, and ≥22 mEq/L) at baseline and the daily study medication dose administered after titration visit (0 g corresponding to placebo, 1.5 g, 3 g, and 4.5 g).

### Statistical Analysis

The analysis follows an exploratory superiority framework to compare the SB and placebo group and was defined in a statistical analysis plan. The sample size calculation was tailored to the primary analysis.^10^ All statistical tests followed a Fisherian significance testing framework. *P*-values are considered exploratory and categorized into levels of evidence against the null hypothesis: no (*P* > 0.1), weak (0.01< *P* ≤ 0.1), substantial (0.001< *P* ≤ 0.01), and strong evidence (*P* ≤ 0.001).^26^ To complement Fisherian inference with a Neyman–Pearson perspective, Holm-adjusted *P*-values are reported for treatment effects on primary outcomes. Baseline characteristics are summarized using descriptive statistics: continuous variables as means ± SD or medians (IQR, Q1-Q3), and categorical variables, including missing values, as counts (%). Percentage changes in concomitant antihypertensive medication (number and dosage) and study medication dosage (% of patients, mean ± SD per visit) are reported descriptively. Missing data, except for lost-to-follow-up, were imputed using 10-fold MICE with predictive mean matching,^27^ and results were pooled according to Rubins rule.^28^ Linear and generalized linear mixed models (LMM and GLMM) incorporating patient-specific random intercepts and fixed effects for treatment group, visit timepoint, and sex (stratification variable) were computed to estimate between-group differences (BGD) and odds ratios (ORs). Baseline adjustment was performed using the ANCOVA method.^29^ The interaction between treatment and time was included if likelihood ratio (LR) tests indicated differential treatment effects. Being hospitalized due to uncontrolled hypertension or volume overload at least once, a binary endpoint, was analyzed using logistic regression. Subgroup analyses were conducted using LMMs to estimate BGDs in subgroups and likelihood ratio tests were used to assess evidence against non-differential treatment effects. Sensitivity analyses were performed on complete case data for primary outcomes to assess robustness. Model assumptions were assessed using diagnostic plots: Q-Q plots for normality of residuals and random effects in LMMs/GLMMs and Tukey-Anscombe plots for homoscedasticity and linearity in LMMs. Logarithmic transformations were applied to address heteroscedasticity where necessary. We report BGD for continuous outcomes and OR for binary outcomes, both with 2-sided 95% confidence intervals (CI). For log-transformed continuous outcomes, treatment effects are also expressed as percentage change on the original scale. Results are visualized using conditional predictions. Subgroup treatment effects are reported as BGDs with 95% CIs. All analyses were conducted in R version 4.3.1 in combination with dynamic reporting to guarantee high standards of reproducibility.

## Results

### Baseline

Patients were recruited from June 12, 2017, to July 10, 2019. Total of 240 patients were included in the modified ITT analysis (119 patients assigned to the SB and 121 to the placebo group).^10^ Baseline characteristics of the study population are shown in Table 1 (missing and log-transformed values in Table S1). Patients had a mean BW of 78.0 ± 17.4 kg in the SB group and 76.2 ± 14.3 kg in the placebo group. The mean percentage of patients with ≤10% in nocturnal systolic ABPM dipping amounted to 19.8% in the SB and 24.4% in the placebo group, respectively. Histories of cardiovascular disease, hypertension, diabetes, and smoking were comparable across groups. Over 80% of patients in both groups had ≥1 antihypertensive medication, RAAS-inhibitors being the most common substance class. Median values of NT-proBNP, plasma renin and aldosterone were comparable between the groups. Median daily sodium excretion was 139.2 mEq (IQR, 118.5-204.6) in the SB and 163.4 mEq (IQR, 117.2-204.5) in the placebo group.

**Table 1:** Baseline Characteristics of the Study Population

Characteristics	Overall	Sodium Bicarbonate	Placebo
	N = 240	n = 119 (49.6%)	n = 121 (50.4%)
**Demographics**			
**Age (y)**	55.5 ± 13.5	55.7 ± 13.2	55.3 ± 13.8
**Sex, male**	167 (69.6%)	82 (68.9%)	85 (70.2%)
**Ethnicity**			
Asian	14 (5.8%)	9 (7.6%)	5 (4.1%)
African	7 (2.9%)	2 (1.7%)	5 (4.1%)
African American	2 (0.8%)	1 (0.8%)	1 (0.8%)
Caucasian	202 (84.2%)	98 (82.4%)	104 (86.0%)
Hispanic	9 (3.8%)	5 (4.2%)	4 (3.3%)
Other	6 (2.5%)	4 (3.4%)	2 (1.7%)
**Body weight (kg)**	77.1 ± 15.9	78.0 ± 17.4	76.2 ± 14.3
**Heart rate (bpm)**	68.7 ± 11.3	68.7 ± 11.3	68.7 ± 11.3
**Office blood pressure (mm Hg)**			
Systolic	132.3 ± 15.3	131.6 ± 15.0	133.0 ± 15.7
Diastolic	80.1 ± 9.9	79.4 ± 9.7	80.8 ± 10.1
MAP	97.5 ± 10.1	96.8 ± 9.7	98.2 ± 10.4
**24 h-ABPM (mm Hg): daytime**			
Systolic	129.0 ± 12.4	129.7 ± 12.7	128.4 ± 12.2
Diastolic	78.0 ± 9.0	78.3 ± 8.5	77.7 ± 9.5
MAP	95.0 ± 8.9	95.4 ± 8.7	94.6 ± 9.1
**24 h-ABPM (mm Hg): nighttime**			
Systolic	123.5 ± 14.8	125.2 ± 15.5	121.8 ± 13.9
Diastolic	72.1 ± 10.0	72.2 ± 9.0	72.0 ± 10.9
MAP	89.2 ± 10.4	89.9 ± 10.0	88.6 ± 10.8
**SBP Dipping**			
Missing	73 (30.4%)	38 (31.9%)	35 (28.9%)
**24 h-ABPM (mm Hg): overall**			
Systolic	127.4 ± 12.1	128.0 ± 12.5	126.8 ± 11.6
Diastolic	76.4 ± 8.7	76.5 ± 8.0	76.3 ± 9.2
MAP	93.4 ± 8.6	93.6 ± 8.4	93.1 ± 8.8
**Comorbidities**			
History of hypertension (treated)	199 (82.9%)	95 (79.8%)	104 (86.0%)
History of diabetes	65 (27.1%)	36 (30.3%)	29 (24.0%)
Cardiovascular disease (total)	105 (43.8%)	54 (45.4%)	51 (42.2%)
Cerebrovascular disease^a^	20 (8.3%)	8 (6.7%)	12 (9.9%)
History of cardiac disease^b^	90 (37.5%)	47 (39.5%)	43 (35.5%)
Peripheral artery disease	16 (6.7%)	7 (5.9%)	9 (7.4%)
Other^c^	8 (3.3%)	3 (2.5%)	5 (4.1%)
History of smoking	93 (38.8%)	49 (41.2%)	44 (36.4%)
**Antihypertensive drugs**			
No medication	34 (14.2%)	21 (17.6%)	13 (10.7%)
Monotherapy	51 (21.3%)	19 (16.0%)	32 (26.5%)
Dual therapy	73 (30.4%)	38 (31.9%)	35 (28.9%)
Triple therapy	44 (18.3%)	23 (19.3%)	21 (16.5%)
More (>3 drugs)	38 (15.8%)	18 (15.1%)	20 (16.5%)
RAAS blockade	150 (62.5%)	71 (59.7%)	79 (65.3%)
Calcium channel blockade	104 (43.3%)	50 (42.0%)	54 (44.6%)
β Blocking agents	126 (52.5%)	68 (57.1%)	58 (47.9%)
Antiadrenergic agents^d^	49 (20.4%)	29 (24.4%)	20 (16.5%)
Diuretics overall	51 (21.3%)	21 (17.7%)	30 (24.8%)
Thiazide diuretics	23 (9.6%)	10 (8.4%)	13 (10.7%)
Loop diuretics	27 (11.3%)	12 (10.1%)	15 (12.4%)
Potassium-sparing diuretics	5 (2.1%)	1 (0.8%)	4 (3.3%)
**Other relevant drugs**			
Lipid modifying agents^e^	146 (60.8%)	73 (61.3%)	73 (60.3%)
Platelet aggregation inhibitors excl. Heparin	81 (33.8%)	42 (35.3%)	39 (32.2%)
Acetylsalicylic acid (ASA)	76 (31.7%)	40 (33.6%)	36 (29.8%)
Antithrombotic agents excl. platelet aggregation inhibitors	19 (7.9%)	8 (6.7%)	11 (9.1%)
**Analytics**			
Serum sodium (mEq/L)	139.0 (138.0-141.0)	139.0 (137.0-141.0)	139.0 (138.0-141.0)
Serum potassium (mEq/L)	4.3 (4.0-4.6)	4.2 (4.0-4.6)	4.3 (3.9-4.6)
NT-proBNP (pg/mL)	221.5 (107.0-529.0)	214.0 (121.0-538.0)	226.0 (94.0-529.0)
Plasma renin (mU/L)	52.2 (18.6-161.5)	46.6 (18.7-137.7)	63.6 (18.1-180.3)
Plasma aldosterone (ng/L)	105.5 (67.7-148.5)	106.5 (68.2-149.0)	103.0 (67.2-148.0)
Aldosterone-to-renin ratio (ng/L per mU/L)	1.7 (0.5-6.9)	2.1 (0.6-6.9)	1.6 (0.5-6.6)
24 h-urine sodium (mEq/24 h)	151.8 (118.0-204.6)	139.2 (118.5-204.6)	163.4 (117.2-204.5)
Sodium/creatinine ratio	11.2 (7.3-16.7)	9.8 (6.5-16.0)	11.8 (8.2-17.6)
24 h-urine potassium (mEq/24 h)	55.2 (43.1-66.2)	58.9 (44.0-67.8)	52.3 (42.5-61.8)
Potassium/creatinine ratio	4.4 (3.3-5.9)	4.6 (3.4-5.9)	4.3 (3.2-5.9)

*Note:* Patient characteristics of the intention-to-treat population assessed at baseline. Continuous variables are reported as means ± standard deviations or medians (interquartile range) and categorical variables as counts N (%). Percentages of missing and log-transformed values can be found in Table S1.

Abbreviations: ABPM, ambulatory blood pressure monitoring; SBP dipping, systolic blood pressure dipping (≥10% decrease in nighttime SBP vs daytime SBP); RAAS, renin-angiotensin-aldosterone system.

a Cerebrovascular disease (stroke, carotid stenosis, vertebral and intracranial stenosis, vascular malformations, intracerebral aneurysms, subarachnoid hemorrhage, and vascular diseases of the eye).

b History of cardiac disease (coronary, hypertensive, valvular, arrhythmogenic and congenital heart disease, coronary sclerosis, and any kind of cardiomyopathy).

c Other (generalized and visceral arteriosclerosis and dilatative arteriopathies).

d Antiadrenergic agents (includes α-adrenoreceptor antagonists used in benign prostatic hypertrophy and tizanidine).

e Lipid modifying agents (includes HMG CoA reductase inhibitors, fibrates, bile acid sequestrants, nicotinic acid and derivatives, and other lipid modifying agents and combinations).

### Variation of Treatment Effects Over Time

The interaction between time and treatment was excluded from all following models, as there was no evidence against a time-constant treatment effect for all primary and secondary outcomes (Tables 2 and 3).

**Table 2:** Treatment Effects of Sodium Bicarbonate on Primary Outcomes

Primary Outcomes	Estimate	Lower 95% CI	Upper 95% CI	Unadjusted *P*-value	Holm-adjusted *P*-value	LR-test *P-*value
Body weight (kg)	1.226	−0.178	2.63	0.09	0.45	0.92
Log-transformed NT-proBNP	−0.026	−0.213	0.161	0.78	1.00	0.27
Log-transformed plasma renin concentration	−0.123	−0.335	0.089	0.26	0.78	0.57
Log-transformed plasma aldosterone concentration	−0.171	−0.296	−0.046	0.007	0.042	0.57
Log-transformed aldosterone-to-renin ratio	−0.047	−0.278	0.185	0.69	1.00	0.93
Nocturnal systolic blood pressure dipping^a^	2.276	0.883	5.869	0.09	0.45	0.57

*Note:* Treatment effects of sodium bicarbonate on primary outcomes (unit) with 95% confidence intervals, *P*-values (unadjusted and Holm-adjusted) and *P*-values of likelihood ratio tests for the interaction between treatment and time. Estimates are time-constant between-group differences for continuous and time-constant odds ratios for binary outcomes, comparing the sodium bicarbonate to the placebo group, accounting for baseline levels.

Abbreviations: CI, confidence interval; LR, likelihood ratio.

a Nocturnal systolic blood pressure dipping (≥10% decrease in nighttime SBP vs daytime SBP).

**Table 3:** Treatment Effects of Sodium Bicarbonate on Secondary Outcomes

Secondary Outcomes	Estimate	Lower 95% CI	Upper 95% CI	*P*-value	LR-test *P*-value
Serum sodium (mEq/L)	0.509	0.082	0.936	0.02	0.65
Log-transformed sodium excretion in 24 h-urine	0.164	0.073	0.255	< 0.001	0.52
Log-transformed sodium/creatinine ratio in spot urine	0.202	0.111	0.293	< 0.001	0.16
Serum potassium concentration (mEq/L)	**−**0.069	**−**0.137	0.0	0.05	0.39
Log-transformed potassium excretion in 24 h-urine	0.007	**−**0.073	0.087	0.86	0.67
Log-transformed potassium/creatinine ratio in spot urine	0.059	**−**0.012	0.129	0.10	0.84
Office hypertension (≥140/90 mm Hg)	1.475	0.999	2.178	0.05	0.85
Hypertension in overall ambulatory blood pressure monitoring (≥130/80 mm Hg)	1.379	0.637	2.987	0.41	0.81

*Note:* Treatment effects of sodium bicarbonate on secondary outcomes (unit) with 95% confidence intervals, *P*-values and *P*-values of likelihood ratio tests for the interaction between treatment and time. Estimates are time-constant between-group differences for continuous and time-constant odds ratios for binary outcomes, comparing the sodium bicarbonate to the placebo group, accounting for baseline levels.

Abbreviations: CI, confidence interval; LR, likelihood ratio.

### Primary Outcomes

#### Body Weight and NT-proBNP

**Figure 1:**
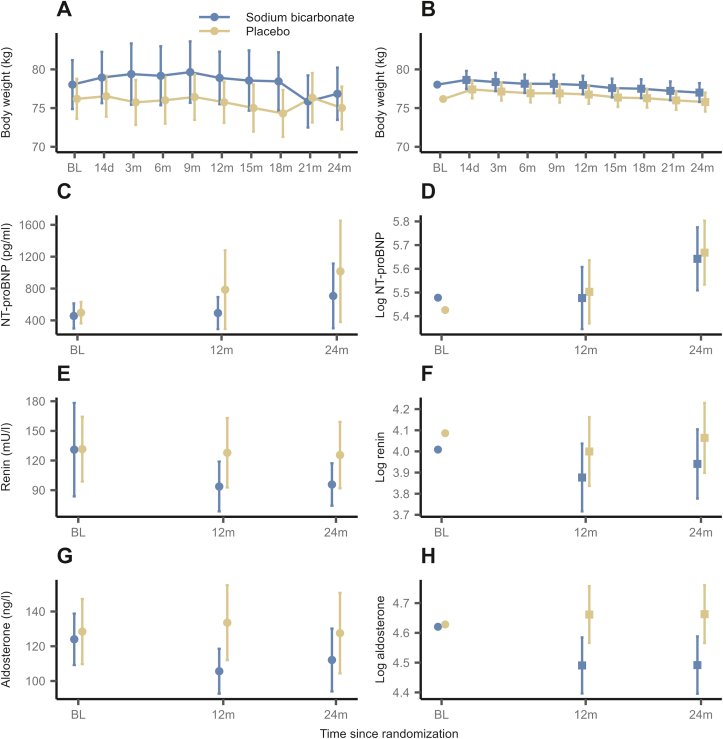
Course of body weight, NT-proBNP, plasma renin, and aldosterone throughout the follow-up period, stratified by treatment group. Assessed (A) and predicted (B) body weight (kg), assessed (pg/mL) (C) and predicted (log-transformed) (D) NT-proBNP, assessed (mU/L) (E) and predicted (log-transformed) (F) plasma renin, assessed (ng/L) (G) and predicted (log-transformed) (H) aldosterone. Points display (raw and predicted) means and errorbars correspond to 95% confidence intervals. Baseline values are displayed as means in panels displaying model predictions, as they were incorporated as covariates in the model.

For BW, observed and model predicted data over 2 years are displayed in Figs 1A and B and changes over time in supplements (Fig S1A). The estimated time-constant difference in BW over 2 years was 1.23 kg (95% CI from −0.18 to 2.63; *P* = 0.09) between the SB and placebo group (Table S2), indicating a trend toward higher BW in the SB group compared with placebo, with weak evidence.

NT-proBNP remained similar in both groups (Figs 1C and D, Fig S1B). The estimated time-constant BGD in log-transformed NT-proBNP was −0.03 (95% CI: −0.21 to 0.16; *P* = 0.78), indicating no evidence for a treatment effect of SB (Table S3).

#### Plasma Renin, Aldosterone, and Aldosterone-to-Renin Ratio

Plasma renin and aldosterone values are shown in Figs 1E-H and Fig S2. We found no evidence for a treatment effect of SB on log-transformed plasma renin (Table 2, Table S4). An estimated time-constant BGD in log-transformed aldosterone of −0.17 (95% CI: −0.3 to −0.05; *P* = 0.007) was identified (Table S5), corresponding to a 15.7% decrease in untransformed aldosterone in the SB group versus placebo, with substantial evidence for a treatment effect. The estimated time-constant difference in the log-transformed aldosterone-to-renin ratio between the SB and placebo groups indicated no evidence for a treatment effect (Fig S3; Table 2 and Table S6).

### Nocturnal Systolic Blood Pressure Dipping

**Figure 2:**
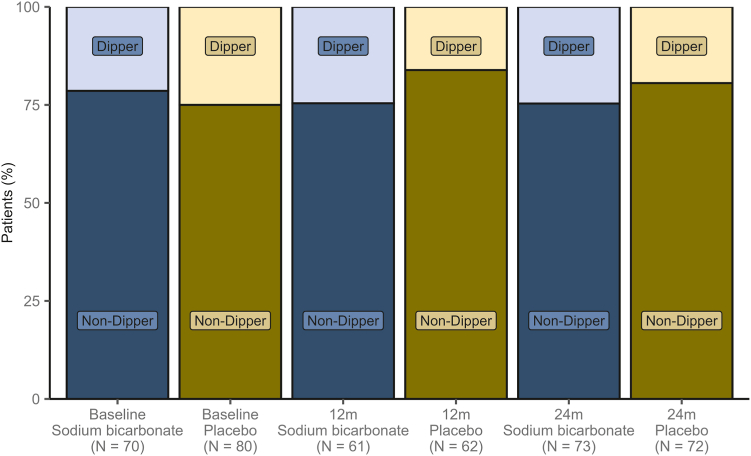
Nocturnal systolic blood pressure dipping. Assessed percentage of patients (Dipper) with nocturnal systolic blood pressure (SBP) dipping ≥ 10% throughout the follow-up period, stratified by treatment group.

Nocturnal SBP dipping is displayed in Fig 2. There was weak evidence for a treatment effect on nocturnal SBP dipping ≥10% in the SB group compared with placebo with an estimated time-constant OR of 2.28 (95% CI: 0.88 to 5.87; *P* = 0.09) (Table S7).

#### Antihypertensive Medication and Diuretics

Changes in the number of antihypertensive agents (including diuretics) and diuretics (separately) over the study period were comparable between the 2 groups (Fig S4; Tables 4 and 5). Changes in total dosages compared to baseline of antihypertensives stratified by substance class were similar in both groups (Table 5). Regarding the change in total dosage of diuretics, we observed a similar decrease in thiazide and increase in loop diuretic dosage in both groups.

**Table 4:** Patients with Change in Number of Antihypertensive and Diuretic Agents

Medication	Baseline to 12 months follow-up		12 to 24 months follow-up	
	SB (n = 106)	Placebo (n = 102)	SB (n = 97)	Placebo (n = 96)
**Number of antihypertensives**				
Increase	13 (12.3%)	19 (18.6%)	11 (11.3%)	12 (12.5%)
Decrease	11 (10.3%)	17 (16.7%)	11 (11.3%)	13 (13.5%)
No Change	82 (77.4%)	66 (64.7%)	75 (77.4%)	71 (74.0%)
**Number of diuretics**				
Increase	6 (5.7%)	5 (4.9%)	4 (4.1%)	7 (7.3%)
Decrease	4 (3.8%)	6 (5.9%)	3 (3.1%)	4 (4.2%)
No Change	96 (90.6%)	91 (89.2%)	90 (92.8%)	85 (88.5%)

*Note:* Number (percentage) of patients with an increase, no change, or decrease in number of antihypertensives (including diuretics) and diuretics (separately) at 12-months and 24-months follow-up compared with baseline (at 12 months) or to 12 months (after 24 months), stratified by treatment group.

Abbreviations: SB, sodium bicarbonate.

**Table 5:** Numbers and Dosages of Antihypertensive and Diuretic Agents

Medication	Baseline		12 months follow-up		24 months follow-up	
	SB	Placebo	SB	Placebo	SB	Placebo
Mean number of antihypertensives	2.07 ± 1.48	2.07 ± 1.34	2.07 ± 1.47	2.06 ± 1.30	1.99± 1.45	2.01 ± 1.25
Mean number of diuretics	0.20 ± 0.48	0.26 ± 0.48	0.25± 0.53	0.23 ± 0.55	0.24 ± 0.52	0.22 ± 0.44
**Dosage of antihypertensives compared with baseline**						
RAAS blockade	100%	100%	81.2%	74.8%	75.5%	65.6%
Calcium channel blockade	100%	100%	74.0%	66.5%	56.4%	49.5%
β Blocking agents	100%	100%	91.6%	86.1%	76.2%	89.1%
Antiadrenergic agents	100%	100%	35.0%	91.3%	40.1%	106.5%
**Dosage of diuretics compared with baseline**						
Thiazide diuretics	100%	100%	92.9%	52.8%	62.8%	49.1%
Loop diuretics	100%	100%	103.3%	105.2%	148.3%	147.0%
Potassium-sparing diuretics	100%	100%	150.0%	64.5%	250.0%	8.1%

*Note:* Mean number of antihypertensives (including diuretics) and diuretics (separately) at baseline, 12-months, and 24-months follow-up, summarized using means ± standard deviations and dosage in percent of antihypertensives and diuretics according to substance class (RAAS blockade, calcium channel blockade, β blocking agents, antiadrenergic agents, thiazide diuretics, loop diuretics, and potassium-sparing diuretics), at 12-months and 24-months follow-up compared with baseline (100%), stratified by treatment group. SB, sodium bicarbonate; RAAS, renin-angiotensin-aldosterone system.

### Secondary Outcomes

#### Sodium

**Figure 3:**
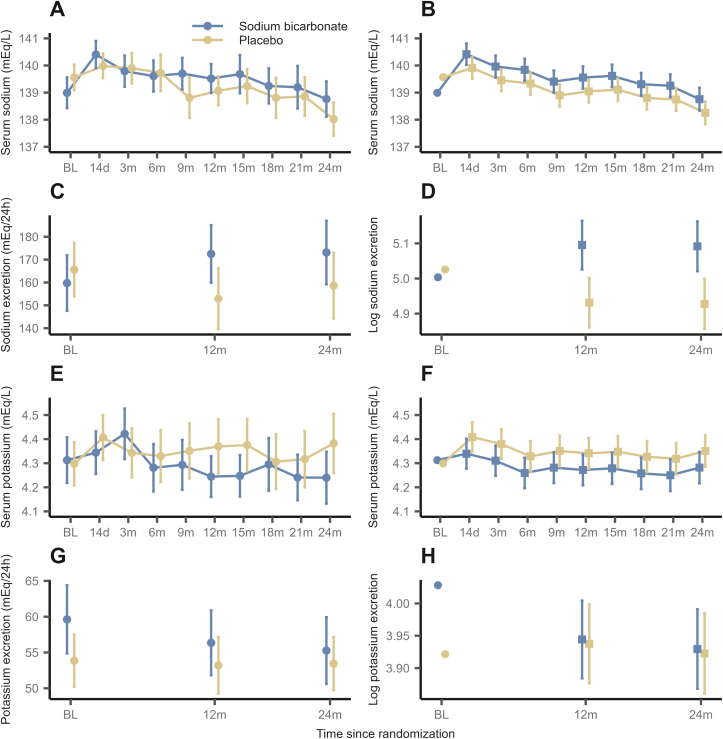
Course of serum sodium, 24 hour-sodium excretion, serum potassium and 24 hour-potassium excretion throughout the follow-up period, stratified by treatment group. Assessed (A) and predicted (B) serum sodium (mEq/L), assessed (mEq/24 h) (C) and predicted (log-transformed) (D) 24 hour-sodium excretion, assessed (E) and predicted (F) serum potassium (mEq/L), assessed (mEq/24 h) (G) and predicted (log-transformed) (H) 24 hour-potassium excretion. Points display (raw and predicted) means and errorbars display 95% confidence intervals. Baseline values are displayed as means in panels displaying model predictions, as they were incorporated as covariates in the model.

In the SB group, the mean SB dosage was 3.00 ± 1.02 g after titration visit and 2.79 ± 1.08 g after 24 months and relatively stable over the course of the study (Fig S5; Tables S8-9). In Figs 3A-D, serum sodium and 24-hour sodium excretion are illustrated. The estimated time-constant BGD of serum sodium was 0.51 mEq/L (95% CI: 0.08 to 0.94; *P* = 0.02), indicating weak evidence for higher levels in the SB group (Table 3). An increase of 17.8% in untransformed 24 hour-sodium excretion in the SB group was detected. In spot urine, similar differences were observed for log-transformed sodium/creatinine ratio, corresponding to an increase of 22.4% in the sodium/creatinine ratio in the SB group (Fig S6; Table 3). Summaries of LMMs are displayed in Tables S10-12.

#### Potassium

There was weak evidence for a treatment effect of SB on serum potassium, as the estimated time-constant BGD between SB and placebo was −0.07 mEq/L (95% CI: −0.14 to 0; *P* = 0.05). We found no evidence for a treatment effect of SB on log-transformed potassium excretion (Figs 3E-H, Fig S7; Table 3, Tables S13-15).

#### Hypertension and Hospitalizations

From 14 days to 24 months, the estimated time-constant OR for OBP ≥140/90 mm Hg between groups was 1.48 (95% CI: 1 to 2.18; *P* = 0.05), indicating a trend toward higher chances of hypertensive OBP measurements in the SB group, with weak evidence. The time-constant estimated OR for overall ABPM ≥130/80 mm Hg showed no evidence for a treatment effect of SB (Table 3, Tables S16-17).

There was no evidence for differences in chances for hospitalizations due to uncontrolled hypertension or clinical signs of volume overload at least once during the study period between study arms. The estimated OR comparing the SB to the placebo group was 3.1 (95% CI: 0.32 to 30.26; *P* = 0.3). In the SB group, 3 patients were hospitalized at least once while it was 1 patient in the placebo group, all due to conditions with clinical signs of volume overload and none due to uncontrolled hypertension.

#### Subgroup and Sensitivity Analyses

Subgroup analyses of the primary outcomes BW, log-transformed NT-proBNP, plasma renin, aldosterone, and aldosterone-to-renin ratio showed no major differential effect of SB across subgroups defined according to eGFR, serum bicarbonate concentration, and number of antihypertensive medications (including diuretics) at baseline and dosage of the study medication administered after titration visit (Figs S8-13; Tables S18-23). A sensitivity analysis of all primary outcomes confirmed the robustness of the results (Table S24).

## Discussion

Contrary to safety concerns, SB therapy did not result in clinically relevant volume retention in KTRs with MA. This is the first large study to systematically assess these potential adverse effects in this population. In this exploratory analysis of a randomized, placebo-controlled trial, SB treatment seemed to decrease plasma aldosterone concentrations without affecting plasma renin and NT-proBNP levels. Although we saw a minor trend toward gain of BW and higher chances of increased OBP, these findings did not translate into differences in ABPM or hospitalizations due to uncontrolled hypertension or volume overload. Antihypertensive and diuretic use were comparable between groups. Serum sodium concentration and urinary sodium excretion were higher in the SB group, the latter confirming increased sodium exposure of SB treated individuals. Overall, our findings do not support a negative effect of SB on volume status in this population, though subtle hemodynamic alterations merit further mechanistic investigation.

In our trial, patients receiving SB faced an additional daily sodium intake of 411-1,233 mg, constituting 11.8%-35.4% of typical daily sodium intake in the Swiss population,^30^ comparable to other European countries.^31^ Sodium overload can drive extracellular volume expansion and BP elevation with subsequent pressure natriuresis.^32^ While, with weak evidence, SB administration showed trends toward higher BW in our KTR population, Yang et al^8^ reported no significant difference between the SB and placebo group in nontransplant patients with CKD. We cannot exclude that the modest increase in BW in the treatment group reflects higher muscle mass following correction of MA, as body composition markers were not assessed in the trial.^2^ Immunosuppressive regimens were unlikely to have influenced the results, as they were similar between groups (>90% receiving calcineurin inhibitors and 40%-45% corticosteroids).^10^ NT-proBNP remained similar in both groups, aligning with findings in nontransplant patients with CKD.^33^ This is reassuring, as NT-proBNP predicts cardiovascular morbidity and mortality in a general population with stable coronary heart disease.^34^ Overall, SB did not appear to cause clinically relevant fluid retention, aligning with the main study, where clinical signs of volume overload as adverse event of special interest occurred similarly across the groups (SB group 6% vs placebo group 8%).^10^

The reported marked suppression of aldosterone in SB-treated patients may partly reflect a physiological reaction to sodium intake^35^ but also a response to MA correction because MA upregulates circulating and kidney intrinsic RAAS to increase net acid excretion.^1^^,^^36^, ^37^, ^38^ Aligning with our results, trends toward lower plasma aldosterone were observed in SB-treated nontransplant patients with CKD.^37^ Given concerns about inaccuracy in measurements due to renin cryoactivation at −20 °C,^39^ we found no discrepancies across the 3 centers (Fig S14).

Salt sensitivity in patients with CKD^40^^,^^41^ and BP reduction by dietary salt restriction^35^ is well described. Because of nephron loss, excessive dietary sodium cannot be excreted adequately, causing higher BP, especially during nighttime, inhibiting physiological dipping.^42^^,^^43^ Unlike SB, dietary salt contains chloride as its accompanying anion, which may play a crucial yet incompletely understood role in salt sensitive hypertension.^44^ Evidence from human and animal studies indicates that NaCl raises BP more than SB or other, nonchloride sodium salts.^45^, ^46^, ^47^, ^48^, ^49^ However, without dietary NaCl restriction, additional sodium may increase BP and sodium retention in CKD regardless of its complementary anion.^11^ In the primary analysis of the Preserve-Transplant Study, the adverse event of special interest newly developed or worsening of preexisting hypertension (9% vs 11%) occurred similarly across groups. Median daytime ABPM and regularly measured OBP showed no significant differences between groups.^10^ To complement these results, we further investigated BP behavior. Surprisingly, the estimated chance of nocturnal SBP dipping, reflecting a healthy circadian BP rhythm, was higher in the SB group, though with weak evidence. SB recipients showed weak evidence for higher chances of hypertension in OBP, but no effect on overall ABPM. Changes in antihypertensive and diuretic use were similar. Overall, SB had no substantial effect on BP, possibly due to the absence of chloride as its accompanying anion. Previous meta-analyses on SB administration in nontransplant CKD patients have reported conflicting findings, including increased SBP^8^ or diastolic blood pressure and worsened hypertension,^21^ and higher antihypertensive use,^50^ while another found no effect on SBP or antihypertensive use.^20^ Reassuringly, hospitalization rates for uncontrolled hypertension or volume overload did not differ in our KTR trial: 3 SB patients were hospitalized once for volume overload compared, to one placebo patient hospitalized 3 times during follow-up.

There was weak evidence for an increase in serum sodium and strong evidence for an increase in sodium excretion (+17.8% in 24 hour-urine, +22.4% in sodium/creatinine ratio) in SB treated patients, indicating a higher sodium burden compared with placebo. However, unmeasured dietary salt intake limits interpretation. It remains unclear whether all additional sodium was excreted, partially retained or less dietary NaCl was ingested due to suppressed salt appetite. Such a reduction could explain why SB administration did not cause substantial volume retention, maintaining tolerable overall sodium balance, while lower chloride intake may diminish its direct effect on renovascular constriction.^51^ However, daily chloride excretion was similar in both groups, as verified in an additional analysis (*P* = 0.95), and the increase in daily natriuresis corresponds approximately to the additional sodium intake with SB in the treatment group, which argues against a substantial difference in salt consumption between the groups. In our KTR population, the estimated time-constant BGD of serum sodium was 0.51 mEq/L. Even small serum sodium increases may be clinically relevant, as in healthy individuals, a 1 mEq/L rise was associated with a higher risk of hypertension.^52^ Furthermore, in CKD patients, increased sodium excretion, comparable to our study, was associated with increased risk of cardiovascular events.^53^

In KTRs, MA was identified as an independent risk factor for ischemic cardiovascular events and was associated with all-cause mortality,^6^ consistent with findings in nontransplant CKD populations.^54^^,^^55^ Randomized controlled trials powered for hard outcomes are lacking and potential underlying mechanisms are not well understood. MA correction with SB could potentially mitigate cardiovascular risk and adverse sodium effects. Indeed, restoring normal serum bicarbonate levels improved vascular endothelial function in patients with CKD.^56^^,^^57^ Alternative to SB, fruits and vegetables were superior to SB in reducing cardiovascular risk and slowing CKD progression in nontransplant patients with CKD,^58^ while evidence is lacking in KTRs.

This post-hoc analysis is limited by its uncertain statistical power and *P*-values requiring cautious interpretation. Generalizability is limited given our predominantly Caucasian study population, while Black individuals may exhibit greater salt sensitivity.^59^

Strengths include the large sample size, randomized, placebo-controlled, single-blinded, and multicenter design and thorough assessments. ABPM as a gold standard complemented OBP measurements. The robustness of the estimated treatment effects was confirmed in a complete-case sensitivity analysis.

Future research should be powered for cardiovascular outcomes, include dietary sodium tracking, and comprise more diverse KTR populations. Clinically, cautious SB use appears reasonable, particularly in patients with substantial cardiovascular comorbidities until its cardiovascular safety is further established.

In conclusion, while some uncertainty remains, our findings do not indicate clinically relevant adverse effects of SB on volume status in KTRs. Nevertheless, targeted studies are warranted to clarify cardiovascular safety profile of SB and to guide use in higher-risk populations.
