# Supplementary material for: Effect of Sodium Bicarbonate on Indicators of Volume Retention in Metabolic Acidosis After Kidney Transplantation: A Post-Hoc Analysis
Source: Kidney Med. 2026 Feb 11;8(4):101290. doi: 10.1016/j.xkme.2026.101290 (PMC13053739; doi:10.1016/j.xkme.2026.101290)
Supplement: Supplementary File (PDF) — Figures S1-S14; Tables S1-S24. [file mmc1.pdf]

Supplementary Material

Supplemental Tables

Table S 1. Baseline characteristics of the study population in detail. 3
Table S 2. Summary of the linear mixed model for body weight. 7
Table S 3. Summary of the linear mixed model for log-transformed NT-proBNP. 8
Table S 4. Summary of the linear mixed model for log-transformed plasma renin concentration. 9
Table S 5. Summary of the linear mixed model for log-transformed plasma aldosterone concentration. 10
Table S 6. Summary of the linear mixed model for log-transformed aldosterone-to-renin ratio. 11
Table S 7. Summary of the generalized linear mixed model for nocturnal systolic blood pressure dipping. 12
Table S 8. Distribution of administered sodium bicarbonate dosages. 13
Table S 9. Mean sodium bicarbonate dosage over time. 14
Table S 10. Summary of the linear mixed model for serum sodium. 15
Table S 11. Summary of the linear mixed model for log-transformed sodium excretion in 24h urine. 16
Table S 12. Summary of the linear mixed model for log-transformed sodium/creatinine ratio in spot urine. 17
Table S 13. Summary of the linear mixed model for serum potassium concentration. 18
Table S 14. Summary of the linear mixed model for log-transformed potassium excretion in 24h urine. 19
Table S 15. Summary of the linear mixed model for log-transformed potassium/creatinine ratio in spot urine. 20
Table S 16. Summary of the generalized linear mixed model for office hypertension. 21
Table S 17. Summary of the generalized linear mixed model for hypertension in overall ambulatory blood pressure monitoring. 22
Table S 18. Subgroup analysis of body weight. 23
Table S 19. Subgroup analysis of NT-proBNP. 24
Table S 20. Subgroup analysis of plasma renin. 25
Table S 21. Subgroup analysis of plasma aldosterone. 26
Table S 22. Subgroup analysis of aldosterone-to-renin ratio. 27
Table S 23. Subgroup analyses of primary outcomes, stratified by dosage. 28
Table S 24. Sensitivity analysis. 29

Supplemental Figures

Figure S 1. Change of body weight and NT-proBNP from baseline. 30
Figure S 2. Change of plasma renin and aldosterone from baseline. 31
Figure S 3. Assessed and predicted courses of and change in aldosterone-to-renin ratio from baseline. 32
Figure S 4. Mean number of antihypertensive agents throughout the follow-up period, stratified by treatment group. 33
Figure S 5. Administered dosage of sodium bicarbonate. 34
Figure S 6. Course of sodium/creatinine ratio throughout the follow-up period, stratified by treatment group. 35
Figure S 7. Course of potassium/creatinine ratio throughout the follow-up period, stratified by treatment group. 36

Figure S 8. Subgroup analysis of body weight. 37
Figure S 9. Subgroup analysis of NT-proBNP. 38
Figure S 10. Subgroup analysis of plasma renin. 39
Figure S 11. Subgroup analysis of plasma aldosterone. 40
Figure S 12. Subgroup analysis of aldosterone-to-renin ratio. 41
Figure S 13. Subgroup analyses of primary outcomes, stratified by dosage. 42
Figure S 14. Distribution of log-transformed plasma renin across 3 study centers. 43

Table S 1. Baseline characteristics of the study population in detail.

Characteristics	Overall	Sodium Bicarbonate	Placebo
	N=240	N = 119 (49.6%)	N = 121 (50.4%)
Demographics			
Age (years)	55.5 ± 13.5	55.7 ± 13.2	55.3 ± 13.8
Sex, male	167 (69.6%)	82 (68.9%)	85 (70.2%)
Ethnicity			
Asian	14 (5.8%)	9 (7.6%)	5 (4.1%)
African	7 (2.9%)	2 (1.7%)	5 (4.1%)
African American	2 (0.8%)	1 (0.8%)	1 (0.8%)
Caucasian	202 (84.2%)	98 (82.4%)	104 (86.0%)
Hispanic	9 (3.8%)	5 (4.2%)	4 (3.3%)
Other	6 (2.5%)	4 (3.4%)	2 (1.7%)
Missing	1 (0.4%)	0 (0.0%)	1 (0.8%)
Body weight (kg)	77.1 ± 15.9	78.0 ± 17.4	76.2 ± 14.3
Missing	1 (0.4%)	0 (0.0%)	1 (0.8%)
Heart rate (bpm)	68.7 ± 11.3	68.7 ± 11.3	68.7 ± 11.3
Missing	4 (1.7%)	2 (1.7%)	2 (1.7%)
Office blood pressure (mmHg)			
Systolic	132.3 ± 15.3	131.6 ± 15.0	133.0 ± 15.7
Diastolic	80.1 ± 9.9	79.4 ± 9.7	80.8 ± 10.1
MAP	97.5 ± 10.1	96.8 ± 9.7	98.2 ± 10.4
Missing	4 (1.7%)	2 (1.7%)	2 (1.7%)
24h-ABPM (mmHg): daytime			
Systolic	129.0 ± 12.4	129.7 ± 12.7	128.4 ± 12.2
Diastolic	78.0 ± 9.0	78.3 ± 8.5	77.7 ± 9.5
MAP	95.0 ± 8.9	95.4 ± 8.7	94.6 ± 9.1
Missing	61 (25.4%)	33 (27.7%)	28 (23.1%)
24h-ABPM (mmHg): nighttime			
Systolic	123.5 ± 14.8	125.2 ± 15.5	121.8 ± 13.9
Diastolic	72.1 ± 10.0	72.2 ± 9.0	72.0 ± 10.9
MAP	89.2 ± 10.4	89.9 ± 10.0	88.6 ± 10.8
Missing	64 (26.7%)	33 (27.7%)	31 (25.6%)
SBP Dipping			
Yes	37 (22.2%)	16 (19.8%)	21 (24.4%)
Missing	73 (30.4%)	38 (31.9%)	35 (28.9%)
24h-ABPM (mmHg): overall			
Systolic	127.4 ± 12.1	128.0 ± 12.5	126.8 ± 11.6
Diastolic	76.4 ± 8.7	76.5 ± 8.0	76.3 ± 9.2
MAP	93.4 ± 8.6	93.6 ± 8.4	93.1 ± 8.8

Missing	61 (25.4%)	33 (27.7%)	28 (23.1%)
Comorbidities			
History of hypertension (treated)	199 (82.9%)	95 (79.8%)	104 (86.0%)
History of diabetes	65 (27.1%)	36 (30.3%)	29 (24.0%)
Cardiovascular disease (total)	105 (43.8%)	54 (45.4%)	51 (42.2%)
Cerebrovascular disease[1]	20 (8.3%)	8 (6.7%)	12 (9.9%)
History of cardiac disease[2]	90 (37.5%)	47 (39.5%)	43 (35.5%)
Peripheral artery disease	16 (6.7%)	7 (5.9%)	9 (7.4%)
Other[3]	8 (3.3%)	3 (2.5%)	5 (4.1%)
History of smoking	93 (38.8%)	49 (41.2%)	44 (36.4%)
Antihypertensive drugs			
No medication	34 (14.2%)	21 (17.6%)	13 (10.7%)
Monotherapy	51 (21.3%)	19 (16.0%)	32 (26.5%)
Dual therapy	73 (30.4%)	38 (31.9%)	35 (28.9%)
Triple therapy	44 (18.3%)	23 (19.3%)	21 (16.5%)
More (>3 drugs)	38 (15.8%)	18 (15.1%)	20 (16.5%)
RAAS blockade	150 (62.5%)	71 (59.7%)	79 (65.3%)
Calcium channel blockade	104 (43.3%)	50 (42.0%)	54 (44.6%)
Beta blocking agents	126 (52.5%)	68 (57.1%)	58 (47.9%)
Antiadrenergic agents[4]	49 (20.4%)	29 (24.4%)	20 (16.5%)
Diuretics overall	51 (21.3%)	21 (17.7%)	30 (24.8%)
Thiazide diuretics	23 (9.6%)	10 (8.4%)	13 (10.7%)
Loop diuretics	27 (11.3%)	12 (10.1%)	15 (12.4%)
Potassium-sparing diuretics	5 (2.1%)	1 (0.8%)	4 (3.3%)
Other relevant drugs			
Lipid modifying agents[5]	146 (60.8%)	73 (61.3%)	73 (60.3%)
Platelet aggregation inhibitors excl. Heparin	81 (33.8%)	42 (35.3%)	39 (32.2%)
Acetylsalicylic acid (ASA)	76 (31.7%)	40 (33.6%)	36 (29.8%)
Antithrombotic agents excl. platelet aggregation inhibitors	19 (7.9%)	8 (6.7%)	11 (9.1%)
Analytics			
Serum sodium (mEq/l)	139.0 [138.0-141.0]	139.0 [137.0-141.0]	139.0 [138.0-141.0]
Missing	1 (0.4%)	0 (0.0%)	1 (0.8%)

Serum potassium (mEq/l)	4.3 [4.0-4.6]	4.2 [4.0-4.6]	4.3 [3.9-4.6]
Missing	1 (0.4%)	0 (0.0%)	1 (0.8%)
NT-proBNP (pg/ml)	221.5 [107.0-529.0]	214.0 [121.0-538.0]	226.0 [94.0-529.0]
Log-transformed NT-proBNP	5.4 ± 1.2	5.5 ± 1.1	5.4 ± 1.3
Missing	6 (2.5%)	4 (3.4%)	2 (1.7%)
Plasma renin (mU/l)	52.2 [18.6-161.5]	46.6 [18.7-137.7]	63.6 [18.1-180.3]
Log-transformed plasma renin	4.0 ± 1.3	4.0 ± 1.3	4.1 ± 1.3
Missing	4 (1.7%)	1 (0.8%)	3 (2.5%)
Plasma aldosterone (ng/l)	105.5 [67.7-148.5]	106.5 [68.2-149.0]	103.0 [67.2-148.0]
Log-transformed plasma aldosterone	4.6 ± 0.6	4.6 ± 0.6	4.6 ± 0.6
Missing	4 (1.7%)	1 (0.8%)	3 (2.5%)
Aldosterone-to-renin ratio (ng/l per mU/l)	1.7 [0.5-6.9]	2.1 [0.6-6.9]	1.6 [0.5-6.6]
Missing	5 (2.1%)	1 (0.8%)	4 (3.3%)
24h urine sodium (mEq/24h)	151.8 [118.0-204.6]	139.2 [118.5-204.6]	163.4 [117.2-204.5]
Log-transformed 24h urine sodium	5.0 ± 0.4	5.0 ± 0.4	5.0 ± 0.4
Missing	15 (6.3%)	10 (8.4%)	5 (4.1%)
Sodium/creatinine ratio	11.2 [7.3-16.7]	9.8 [6.5-16.0]	11.8 [8.2-17.6]
Log-transformed sodium/creatinine ratio	2.4 ± 0.7	2.3 ± 0.7	2.5 ± 0.7
Missing	4 (1.7%)	4 (3.4%)	0 (0.0%)
24h urine potassium (mEq/24h)	55.2 [43.1-66.2]	58.9 [44.0-67.8]	52.3 [42.5-61.8]
Log-transformed 24h urine potassium	4.0 ± 0.4	4.0 ± 0.4	3.9 ± 0.4
Missing	16 (6.7%)	10 (8.4%)	6 (5.0%)
Potassium/creatinine ratio	4.4 [3.3-5.9]	4.6 [3.4-5.9]	4.3 [3.2-5.9]
Log-transformed potassium/creatinine ratio	1.5 ± 0.5	1.5 ± 0.4	1.5 ± 0.5
Missing	4 (1.7%)	4 (3.4%)	0 (0.0%)

Patient characteristics of the intention-to-treat population assessed at baseline. Continuous variables are reported as means ± standard deviations or medians [interquartile range] and categorical variables as counts N (%).[1] Cerebrovascular disease (stroke, carotid stenosis, vertebral and intracranial stenosis, vascular malformations, intracerebral aneurysms, subarachnoid hemorrhage, vascular diseases of the eye). [2] History of cardiac disease

(coronary, hypertensive, valvular, arrhythmogenic and congenital heart disease, coronary sclerosis, any kind of cardiomyopathy). [3] Other (generalized and visceral arteriosclerosis and dilatative arteriopathies). [4] Antiadrenergic agents (includes alpha-adrenoreceptor antagonists used in benign prostatic hypertrophy and tizanidine). [5] Lipid modifying agents (includes HMG CoA reductase inhibitors, fibrates, bile acid sequestrants, nicotinic acid and derivatives, other lipid modifying agents and combinations). ABPM, ambulatory blood pressure monitoring; SBP, systolic blood pressure dipping (≥10% decrease in nighttime SBP vs. daytime SBP); RAAS, renin-angiotensin-aldosterone system.

Table S 2. Summary of the linear mixed model for body weight.

Variable	Estimate	Lower 95% CI	Upper 95% Ci	P-value
Intercept	10.547	6.949	14.145	<0.001
**Sodium bicarbonate**	**1.226**	**-0.178**	**2.63**	**0.09**
Visit at 3 months	-0.274	-1.765	1.218	0.72
Visit at 6 months	-0.492	-1.975	0.991	0.52
Visit at 9 months	-0.508	-2.109	1.094	0.53
Visit at 12 months	-0.656	-1.908	0.596	0.30
Visit at 15 months	-1.051	-2.397	0.295	0.13
Visit at 18 months	-1.136	-2.605	0.334	0.13
Visit at 21 months	-1.416	-2.889	0.058	0.06
Visit at 24 months	-1.632	-2.9	-0.365	0.012
Adjusted for				
Baseline	0.837	0.789	0.884	<0.001
Male sex	3.041	1.409	4.672	<0.001

Estimates with 95% confidence intervals (CI) and p-values for body weight (kg) are displayed. The estimate for the sodium bicarbonate group is the estimated time-constant mean difference to the placebo group, accounting for body weight at baseline.

Table S 3. Summary of the linear mixed model for log-transformed NT-proBNP.

Variable	Estimate	Lower 95% CI	Upper 95% Ci	P-value
Intercept	0.777	0.266	1.287	0.003
**Sodium bicarbonate**	**-0.026**	**-0.213**	**0.161**	**0.78**
Visit at 24 months	0.165	0.062	0.269	0.002
Adjusted for				
Baseline	0.872	0.789	0.955	<0.001
Male sex	0.008	-0.201	0.216	0.94

Estimates with 95% confidence intervals (CI) and p-values for log-transformed NT-proBNP are displayed. The estimate for the sodium bicarbonate group is the estimated time-constant mean difference to the placebo group, accounting for baseline NT-proBNP concentration.

Table S 4. Summary of the linear mixed model for log-transformed plasma renin concentration.

Variable	Estimate	Lower 95% CI	Upper 95% Ci	P-value
Intercept	1.158	0.773	1.543	<0.001
**Sodium bicarbonate**	**-0.123**	**-0.335**	**0.089**	**0.26**
Visit at 24 months	0.064	-0.073	0.202	0.36
Adjusted for				
Baseline	0.636	0.548	0.724	<0.001
Male sex	0.399	0.153	0.644	0.001

Estimates with 95% confidence intervals (CI) and p-values for log-transformed plasma renin are displayed. The estimate for the sodium bicarbonate group is the estimated time-constant mean difference to the placebo group, accounting for baseline plasma renin.

Table S 5. Summary of the linear mixed model for log-transformed plasma aldosterone concentration.

Variable	Estimate	Lower 95% CI	Upper 95% Ci	P-value
Intercept	2.49	1.964	3.016	<0.001
**Sodium bicarbonate**	**-0.171**	**-0.296**	**-0.046**	**0.007**
Visit at 24 months	0.001	-0.088	0.091	0.98
Adjusted for				
Baseline	0.479	0.372	0.585	<0.001
Male sex	-0.029	-0.169	0.11	0.68

Estimates with 95% confidence intervals (CI) and p-values for log-transformed plasma aldosterone are displayed. The estimate for the sodium bicarbonate group is the estimated time-constant mean difference to the placebo group, accounting for baseline plasma aldosterone.

Table S 6. Summary of the linear mixed model for log-transformed aldosterone-to-renin ratio.

Variable	Estimate	Lower 95% CI	Upper 95% Ci	P-value
Intercept	0.673	0.396	0.95	<0.001
**Sodium bicarbonate**	**-0.047**	**-0.278**	**0.185**	**0.69**
Visit at 24 months	-0.063	-0.222	0.095	0.43
Adjusted for				
Baseline	0.571	0.487	0.656	<0.001
Male sex	-0.469	-0.738	-0.201	<0.001

Estimates with 95% confidence intervals (CI) and p-values for log-transformed aldosterone-to-renin ratio displayed. The estimate for the sodium bicarbonate group is the estimated time-constant mean difference to the placebo group, accounting for baseline values.

Table S 7. Summary of the generalized linear mixed model for nocturnal systolic blood pressure dipping.

Variable	Estimate	Lower 95% CI	Upper 95% Ci	P-value
Intercept	0.033	0.006	0.192	<0.001
**Sodium bicarbonate**	**2.276**	**0.883**	**5.869**	**0.09**
Visit at 24 months	1.25	0.574	2.723	0.57
Adjusted for				
Baseline	5.713	1.713	19.052	0.005
Male sex	2.832	0.892	8.989	0.08

Estimates with 95% confidence intervals (CI) and p-values for nocturnal systolic blood pressure dipping are displayed. The estimate for the sodium bicarbonate group is the estimated time-constant odds ratio comparing the sodium bicarbonate group to the placebo group.

Table S 8. Distribution of administered sodium bicarbonate dosages.

Visit	0.5g/day	1g/day	1.5g/day	2g/day	3g/day	4.5g/day
Baseline	0%	0%	36%	0%	64%	0%
14 days	0%	0%	23%	0%	54%	23%
3 months	0%	3%	20%	4%	51%	22%
6 months	0%	0%	24%	4%	52%	20%
9 months	0%	1%	21%	5%	53%	20%
12 months	1%	3%	23%	4%	49%	19%
15 months	1%	1%	22%	5%	51%	18%
18 months	1%	1%	23%	4%	51%	20%
21 months	1%	1%	24%	3%	51%	20%
24 months	1%	4%	24%	4%	49%	19%

Patients (%) with administered dosage (g/day) of sodium bicarbonate (study medication) in the sodium bicarbonate group at each visit.

Table S 9. Mean sodium bicarbonate dosage over time.

Visit	Mean	SD
Baseline	2.46	0.72
14 days	3.00	1.02
3 months	2.92	1.06
6 months	2.91	1.02
9 months	2.91	1.01
12 months	2.79	1.08
15 months	2.83	1.04
18 months	2.86	1.06
21 months	2.85	1.07
24 months	2.79	1.08

Mean (SD) dosage (g) of sodium bicarbonate administered in the sodium bicarbonate group stratified by time of visit.

Table S 10. Summary of the linear mixed model for serum sodium.

Variable	Estimate	Lower 95% CI	Upper 95% Ci	P-value
Intercept	77.375	67.243	87.507	<0.001
**Sodium bicarbonate**	**0.509**	**0.082**	**0.936**	**0.02**
Visit at 3 months	-0.453	-0.892	-0.015	0.04
Visit at 6 months	-0.575	-1.04	-0.11	0.02
Visit at 9 months	-1.01	-1.459	-0.561	<0.001
Visit at 12 months	-0.861	-1.283	-0.439	<0.001
Visit at 15 months	-0.801	-1.27	-0.331	<0.001
Visit at 18 months	-1.107	-1.586	-0.628	<0.001
Visit at 21 months	-1.162	-1.634	-0.69	<0.001
Visit at 24 months	-1.659	-2.09	-1.228	<0.001
Adjusted for				
Baseline	0.449	0.376	0.521	<0.001
Male sex	0.155	-0.309	0.619	0.51

Estimates with 95% confidence intervals (CI) and p-values for serum sodium (mEq/l) are displayed. The estimate for the sodium bicarbonate group is the estimated time-constant mean difference to the placebo group, accounting for baseline serum sodium.

Table S 11. Summary of the linear mixed model for log-transformed sodium excretion in 24h urine.

Variable	Estimate	Lower 95% CI	Upper 95% Ci	P-value
Intercept	2.898	2.321	3.475	<0.001
**Sodium bicarbonate**	**0.164**	**0.073**	**0.255**	<0.001
Visit at 24 months	-0.002	-0.068	0.06	0.91
Adjusted for				
Baseline	0.394	0.277	0.51	<0.001
Male sex	0.089	-0.016	0.195	0.10

Estimates with 95% confidence intervals (CI) and p-values for log-transformed 24h sodium excretion are displayed. The estimate for the sodium bicarbonate group is the estimated time-constant mean difference to the placebo group, adjusting for baseline sodium excretion.

Table S 12. Summary of the linear mixed model for log-transformed sodium/creatinine ratio in spot urine.

Variable	Estimate	Lower 95% CI	Upper 95% Ci	P-value
Intercept	1.677	1.47	1.885	<0.001
**Sodium bicarbonate**	**0.202**	**0.111**	**0.293**	**<0.001**
Visit at 6 months	-0.009	-0.124	0.106	0.88
Visit at 9 months	-0.043	-0.151	0.065	0.44
Visit at 12 months	-0.03	-0.136	0.076	0.58
Visit at 15 months	-0.07	-0.193	0.053	0.27
Visit at 18 months	-0.037	-0.157	0.082	0.54
Visit at 21 months	-0.06	-0.18	0.059	0.32
Visit at 24 months	-0.005	-0.112	0.103	0.93
Adjusted for				
Baseline	0.319	0.253	0.384	<0.001
Male sex	-0.039	-0.14	0.061	0.44

Estimates with 95% confidence intervals (CI) and p-values for log-transformed sodium/creatinine ratio are displayed. The estimate for the sodium bicarbonate group is the estimated time-constant mean difference to the placebo group, accounting for baseline values.

Table S 13. Summary of the linear mixed model for serum potassium concentration.

Variable	Estimate	Lower 95% CI	Upper 95% Ci	P-value
Intercept	2.047	1.746	2.348	<0.001
**Sodium bicarbonate**	**-0.069**	**-0.137**	**0.0**	**0.05**
Visit at 3 months	-0.029	-0.101	0.043	0.43
Visit at 6 months	-0.08	-0.151	-0.008	0.03
Visit at 9 months	-0.058	-0.132	0.016	0.12
Visit at 12 months	-0.067	-0.135	0	0.05
Visit at 15 months	-0.061	-0.132	0.01	0.09
Visit at 18 months	-0.082	-0.154	-0.009	0.03
Visit at 21 months	-0.09	-0.164	-0.015	0.02
Visit at 24 months	-0.058	-0.127	0.011	0.10
Adjusted for				
Baseline	0.537	0.468	0.607	<0.001
Male sex	0.073	-0.002	0.147	0.06

Estimates with 95% confidence intervals (CI) and p-values for serum potassium (mEq/l) are displayed. The estimate for the sodium bicarbonate group is the estimated time-constant mean difference to the placebo group, accounting for baseline values.

Table S 14. Summary of the linear mixed model for log-transformed potassium excretion in 24h urine.

Variable	Estimate	Lower 95% CI	Upper 95% Ci	P-value
Intercept	2.106	1.67	2.542	<0.001
**Sodium bicarbonate**	**0.007**	**-0.073**	**0.087**	**0.86**
Visit at 24 months	-0.015	-0.072	0.043	0.61
Adjusted for				
Baseline	0.446	0.332	0.56	<0.001
Male sex	0.085	-0.004	0.175	0.06

Estimates with 95% confidence intervals (CI) and p-values for log-transformed 24h potassium excretion are displayed. The estimate for the sodium bicarbonate group is the estimated time-constant mean difference to the placebo group, adjusting for baseline values.

Table S 15. Summary of the linear mixed model for log-transformed potassium/creatinine ratio in spot urine.

Variable	Estimate	Lower 95% CI	Upper 95% Ci	P-value
Intercept	1.053	0.901	1.205	<0.001
**Sodium bicarbonate**	**0.059**	**-0.012**	**0.129**	**0.10**
Visit at 6 months	-0.008	-0.078	0.062	0.83
Visit at 9 months	-0.012	-0.086	0.062	0.75
Visit at 12 months	-0.043	-0.111	0.026	0.22
Visit at 15 months	-0.036	-0.122	0.049	0.40
Visit at 18 months	-0.055	-0.132	0.023	0.17
Visit at 21 months	-0.008	-0.095	0.078	0.85
Visit at 24 months	-0.014	-0.085	0.057	0.70
Adjusted for				
Baseline	0.373	0.299	0.447	<0.001
Male sex	-0.13	-0.209	-0.051	0.001

Estimates with 95% confidence intervals (CI) and p-values for potassium/creatinine ratio are displayed. The estimate for the sodium bicarbonate group is the estimated time-constant mean difference to the placebo group, accounting for baseline values.

Table S 16. Summary of the generalized linear mixed model for office hypertension.

Variable	Estimate	Lower 95% CI	Upper 95% Ci	P-value
Intercept	0.185	0.104	0.329	<0.001
**Sodium bicarbonate**	**1.475**	**0.999**	**2.178**	**0.05**
Visit at 3 months	1.349	0.84	2.167	0.22
Visit at 6 months	1.324	0.786	2.228	0.29
Visit at 9 months	1.37	0.788	2.382	0.26
Visit at 12 months	1.18	0.721	1.931	0.51
Visit at 15 months	1.273	0.742	2.186	0.38
Visit at 18 months	1.27	0.732	2.203	0.39
Visit at 21 months	0.87	0.528	1.434	0.58
Visit at 24 months	1.573	0.964	2.567	0.07
Adjusted for				
Baseline	2.957	1.964	4.452	<0.001
Male sex	1.012	0.655	1.564	0.96

Estimated odds ratios with 95% confidence intervals (CI) and p-values for office hypertension are displayed. The estimate for the sodium bicarbonate group is the estimated time-constant odds ratio to the placebo group, accounting for baseline office hypertension.

Table S 17. Summary of the generalized linear mixed model for hypertension in overall ambulatory blood pressure monitoring.

Variable	Estimate	Lower 95% CI	Upper 95% Ci	P-value
Intercept	0.52	0.193	1.4	0.20
**Sodium bicarbonate**	**1.379**	**0.637**	**2.987**	**0.41**
Visit at 24 months	0.918	0.476	1.767	0.80
Adjusted for				
Baseline	7.027	2.688	18.371	<0.001
Male sex	0.433	0.177	1.06	0.07

Estimated odds ratios with 95% confidence intervals (CI) and p-values for overall ambulatory blood pressure monitoring are displayed. The estimate for the sodium bicarbonate group is the estimated time-constant odds ratio to the placebo group, accounting for overall ambulatory blood pressure monitoring hypertension at baseline.

Table S 18. Subgroup analysis of body weight.

Subgroups	Treatment Effect	Lower 95% CI	Upper 95% Ci
**eGFR (Baseline)**			
15 to <30 ml/min/1.73m2	0.17	-3.897	4.237
30 to <45 ml/min/1.73m2	0.991	-1.324	3.307
45 to <60 ml/min/1.73m2	1.621	-1.037	4.279
≥60 ml/min/1.73m2	1.52	-1.118	4.157
**Serum bicarbonate (Baseline)**			
≤18 mEq/l	1.46	-2.599	5.519
18 to <20 mEq/l	1.254	-2.494	5.002
20 to <22 mEq/l	1.263	-1.194	3.72
≥22 mEq/l	0.777	-1.434	2.987
**Antihypertensive drugs incl. diuretics (Baseline)**			
No concomitant medication	1.916	-3.372	7.204
Monotherapy	1.799	-1.041	4.639
Dual therapy	1.731	-0.946	4.408
Triple therapy	1.031	-2.338	4.4
More than 3	-3.448	-9.488	2.591

Estimated treatment effects in subgroup analysis for body weight (kg) with 95% confidence intervals (CI). Treatment effects are mean differences between the sodium bicarbonate group and the placebo group. Abbreviation: eGFR, estimated glomerular filtration rate.

Table S 19. Subgroup analysis of NT-proBNP.

Subgroups	Treatment Effect	Lower 95% CI	Upper 95% Ci
**eGFR (Baseline)**			
15 to <30 ml/min/1.73m2	-0.017	-0.599	0.565
30 to <45 ml/min/1.73m2	0.013	-0.315	0.341
45 to <60 ml/min/1.73m2	-0.032	-0.375	0.312
≥60 ml/min/1.73m2	-0.087	-0.47	0.297
**Serum bicarbonate (Baseline)**			
≤18 mEq/l	-0.054	-0.599	0.491
18 to <20 mEq/l	0.08	-0.471	0.631
20 to <22 mEq/l	-0.063	-0.402	0.277
≥22 mEq/l	0.025	-0.286	0.335
**Antihypertensive drugs incl. diuretics (Baseline)**			
No concomitant medication	0.065	-0.591	0.721
Monotherapy	-0.212	-0.59	0.166
Dual therapy	0.025	-0.319	0.368
Triple therapy	-0.209	-0.626	0.209
More than 3	0.264	-0.519	1.046

Estimated treatment effects in subgroup analysis for log-transformed NT-proBNP with 95% confidence intervals (CI). Treatment effects are mean differences between the sodium bicarbonate group and the placebo group. Abbreviation: eGFR, estimated glomerular filtration rate.

Table S 20. Subgroup analysis of plasma renin.

Subgroups	Treatment Effect	Lower 95% CI	Upper 95% Ci
**eGFR (Baseline)**			
15 to <30 ml/min/1.73m2	-0.257	-0.916	0.401
30 to <45 ml/min/1.73m2	-0.131	-0.494	0.232
45 to <60 ml/min/1.73m2	-0.026	-0.433	0.38
≥60 ml/min/1.73m2	-0.197	-0.627	0.233
**Serum bicarbonate (Baseline)**			
≤18 mEq/l	-0.084	-0.733	0.565
18 to <20 mEq/l	-0.284	-0.875	0.308
20 to <22 mEq/l	0.014	-0.368	0.397
≥22 mEq/l	-0.203	-0.537	0.13
**Antihypertensive drugs incl. diuretics (Baseline)**			
No concomitant medication	-0.845	-1.638	-0.051
Monotherapy	0.115	-0.305	0.535
Dual therapy	-0.167	-0.543	0.21
Triple therapy	-0.06	-0.548	0.428
More than 3	-0.219	-1.141	0.703

Estimated treatment effects in subgroup analysis for log-transformed plasma renin with 95% confidence intervals (CI). Treatment effects are mean differences between the sodium bicarbonate group and the placebo group. Abbreviation: eGFR, estimated glomerular filtration rate. Abbreviation: eGFR, estimated glomerular filtration rate.

Table S 21. Subgroup analysis of plasma aldosterone.

Subgroups	Treatment Effect	Lower 95% CI	Upper 95% Ci
**eGFR (Baseline)**			
15 to <30 ml/min/1.73m2	-0.036	-0.41	0.337
30 to <45 ml/min/1.73m2	-0.258	-0.471	-0.045
45 to <60 ml/min/1.73m2	-0.105	-0.333	0.122
≥60 ml/min/1.73m2	-0.171	-0.418	0.076
**Serum bicarbonate (Baseline)**			
≤18 mEq/l	0.034	-0.34	0.409
18 to <20 mEq/l	-0.123	-0.463	0.216
20 to <22 mEq/l	-0.296	-0.517	-0.075
≥22 mEq/l	-0.132	-0.328	0.064
**Antihypertensive drugs/diuretics (Baseline)**			
No concomitant medication	-0.51	-0.987	-0.034
Monotherapy	-0.048	-0.313	0.218
Dual therapy	-0.255	-0.482	-0.029
Triple therapy	-0.024	-0.315	0.266
More than 3	-0.392	-0.94	0.156

Estimated treatment effects in subgroup analysis for log-transformed plasma aldosterone with 95% confidence intervals (CI). Treatment effects are mean differences between the sodium bicarbonate group and the placebo group.

Table S 22. Subgroup analysis of aldosterone-to-renin ratio.

Subgroups	Treatment Effect	Lower 95% CI	Upper 95% Ci
**eGFR (Baseline)**			
15 to <30 ml/min/1.73m2	0.238	-0.496	0.971
30 to <45 ml/min/1.73m2	-0.155	-0.553	0.243
45 to <60 ml/min/1.73m2	-0.089	-0.533	0.355
≥60 ml/min/1.73m2	0.068	-0.401	0.537
**Serum bicarbonate (Baseline)**			
≤18 mEq/l	0.04	-0.66	0.741
18 to <20 mEq/l	0.185	-0.46	0.829
20 to <22 mEq/l	-0.284	-0.7	0.133
≥22 mEq/l	0.063	-0.302	0.428
**Antihypertensive drugs incl. diuretics (Baseline)**			
No concomitant medication	0.35	-0.571	1.271
Monotherapy	-0.17	-0.672	0.331
Dual therapy	-0.063	-0.5	0.374
Triple therapy	0.041	-0.516	0.598
More than 3	-0.133	-1.189	0.924

Estimated treatment effects in subgroup analysis for the log-transformed ratio between plasma aldosterone to renin with 95% confidence intervals (CI). Treatment effects are mean differences between the sodium bicarbonate group and the placebo group. Abbreviation: eGFR, estimated glomerular filtration rate.

Table S 23. Subgroup analyses of primary outcomes, stratified by dosage.

Subgroups	Treatment Effect	Lower 95% CI	Upper 95% Ci
**Body weight (kg)**			
1.5g/day	-0.893	-3.384	1.599
3g/day	1.352	-0.364	3.068
4.5g/day	3.182	0.848	5.516
**Log-transformed NT-proBNP**			
1.5g/day	0.057	-0.297	0.411
3g/day	-0.035	-0.259	0.189
4.5g/day	-0.082	-0.426	0.262
**Log-transformed plasma renin**			
1.5g/day	-0.035	-0.394	0.323
3g/day	-0.117	-0.383	0.149
4.5g/day	-0.23	-0.619	0.158
**Log-transformed plasma aldosterone**			
1.5g/day	-0.157	-0.367	0.053
3g/day	-0.184	-0.334	-0.035
4.5g/day	-0.148	-0.373	0.077
**Log-transformed aldosterone-to-renin ratio**			
1.5g/day	-0.10	-0.48	0.28
3g/day	-0.07	-0.36	0.21
4.5g/day	0.08	-0.33	0.49

Estimated effects of sodium bicarbonate dosage assigned at the dosage titration visit, compared to the placebo group, on primary outcomes. Effects are mean differences from a dosage group to the placebo group, presented with 95% confidence intervals (CI) and computed with linear mixed models.

Table S 24. Sensitivity analysis.

Outcome	Main analysis	95% CI	Sensitivity analysis	95% CI
Body weight (kg)	1.226	-0.178 to 2.63	0.807	0.001 to 1.613
Log-transformed NT-proBNP	-0.026	-0.213 to 0.161	-0.049	-0.218 to 0.121
Log-transformed plasma renin	-0.123	-0.335 to 0.089	-0.108	-0.321 to 0.105
Log-transformed plasma aldosterone	-0.171	-0.296 to -0.046	-0.188	-0.314 to -0.062
**Log-transformed aldosterone-to-renin ratio**	-0.047	-0.278 to 0.185	-0.093	-0.328 to 0.142
SBP dipping (%)	2.276	00.883 to 5.869	2.273	0.974 to 5.302

Treatment effects from the main analysis, performed on multiply imputed data, and from the sensitivity analysis, performed on complete cases only, with 95% confidence intervals (CI). Abbreviation: SBP, systolic blood pressure.

Supplemental Figures

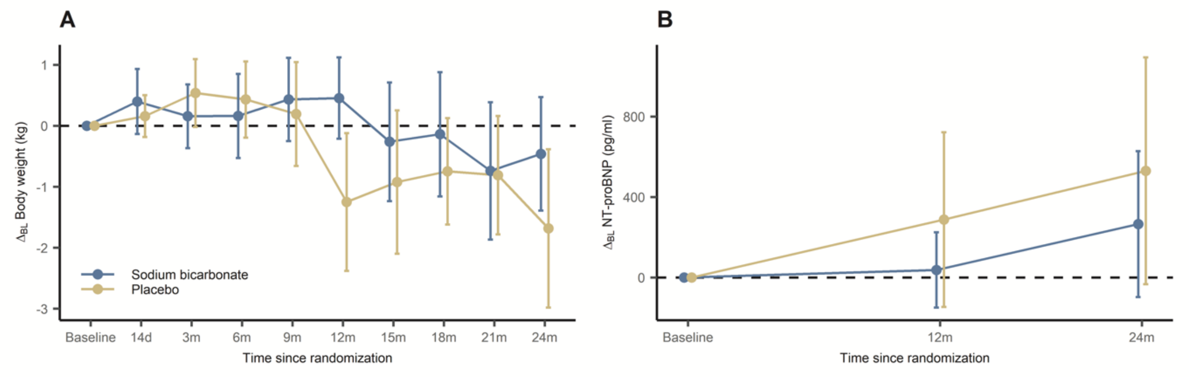

Figure S 1. Change of body weight and NT-proBNP from baseline. (A) Change of body weight (kg) and (B) NT-proBNP (pg/ml) from baseline displayed as means with 95% confidence intervals.

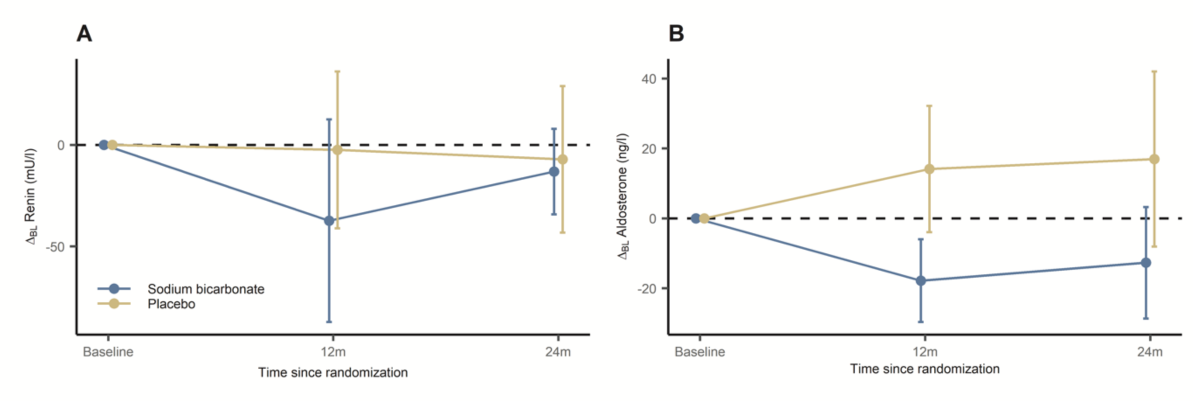

Figure S 2. Change of plasma renin and aldosterone from baseline. (A) Change of plasma renin (mU/l) and (B) aldosterone (ng/l) from baseline (BL) displayed as means with 95% confidence intervals.

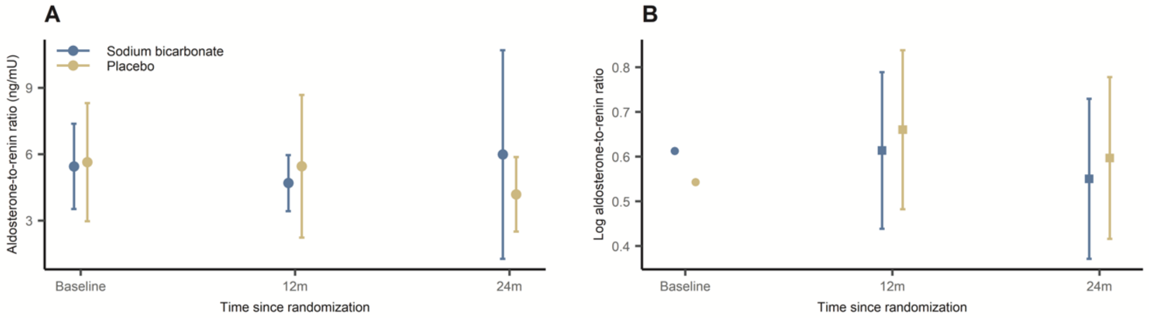

Figure S 3. Assessed and predicted courses of and change in aldosterone-to-renin ratio from baseline. (A) Assessed (ng/l per mU/l) and (B) predicted (log-transformed) aldosterone-to-renin ratio assessed with a linear mixed model throughout the follow-up period, stratified by treatment group. Points display means and errorbars display 95% confidence intervals. Baseline values are displayed as means, as they were incorporated as covariates in the model (B).

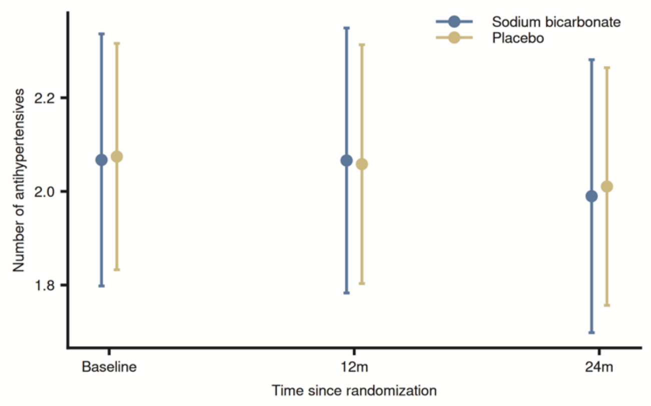

Figure S 4. **Mean number of antihypertensive agents throughout the follow-up period, stratified by treatment group.** Assessed number of concomittant antihypertensive drugs (including diuretics). Points display means and errorbars display 95% confidence intervals.

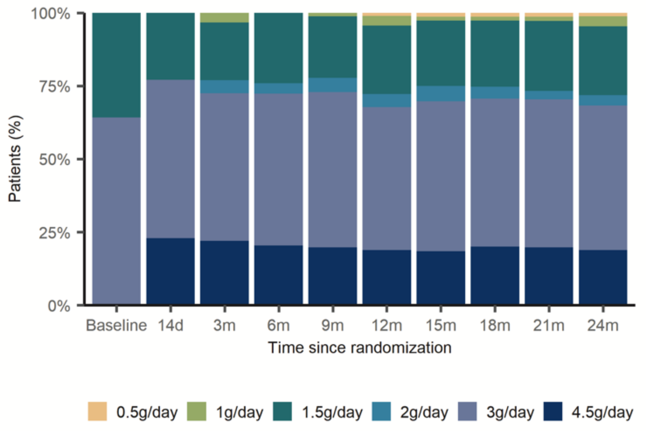

Figure S 5. Administered dosage of sodium bicarbonate. Administered dosage (g/day) of sodium bicarbonate (study medication) in the sodium bicarbonate group throughout the follow-up period.

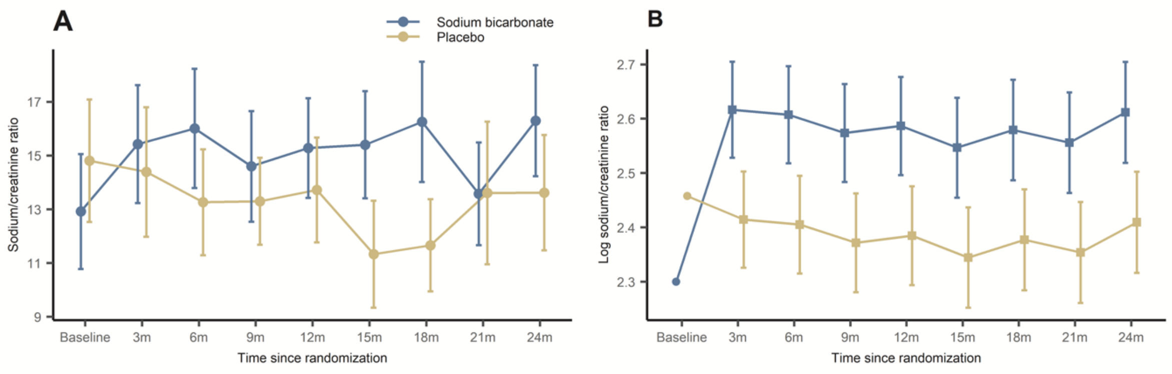

Figure S 6. Course of sodium/creatinine ratio throughout the follow-up period, stratified by treatment group. (A) Assessed sodium/creatinine ratio. Points display means and errorbars display 95% confidence intervals. (B) Predicted log-transformed sodium/creatinine ratio assessed with a linear mixed model. Errorbars correspond to 95% confidence intervals of predictions. Baseline values are displayed as means, as they were incorporated as covariates in the model.

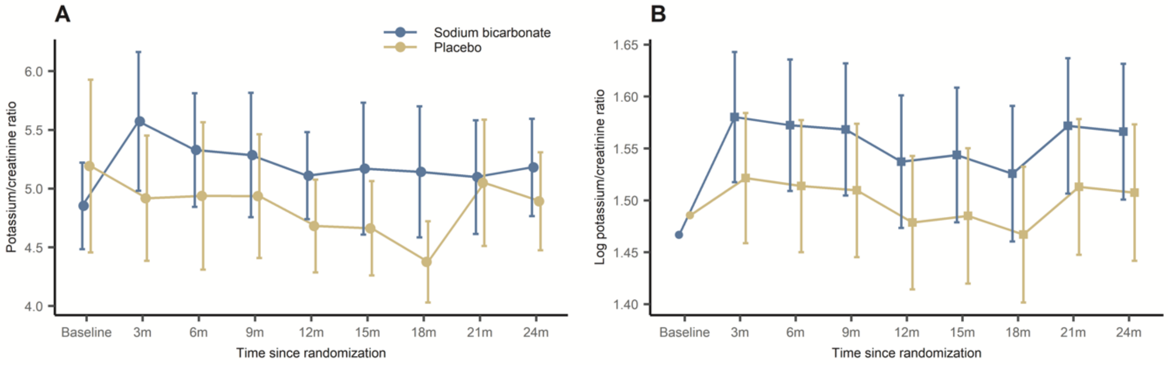

Figure S 7. Course of potassium/creatinine ratio throughout the follow-up, stratified by treatment group. (A) Assessed potassium/creatinine ratio. Points display means and errorbars display 95% confidence intervals. (B) Predicted log-transformed potassium/creatinine ratio assessed with a linear mixed model. Errorbars correspond to 95% confidence intervals of predictions. Baseline values are displayed as means, as they were incorporated as covariates in the model.

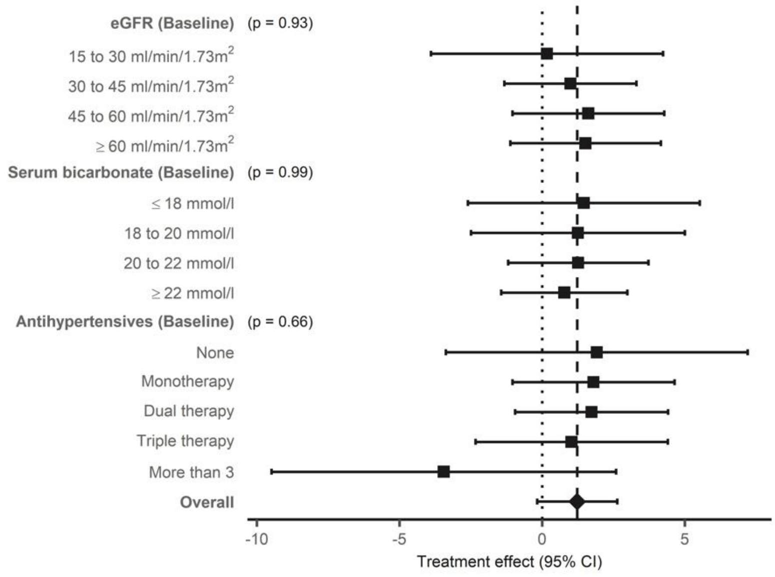

Figure S 8. Subgroup analysis of body weight. Treatment effects with 95% confidence intervals in subgroup analyses for body weight (kg), assessed with linear mixed models. The p-values correspond to the likelihood ratio tests comparing models with and without the interaction between treatment and subgroup. Abbreviation: eGFR, estimated glomerular filtration rate.

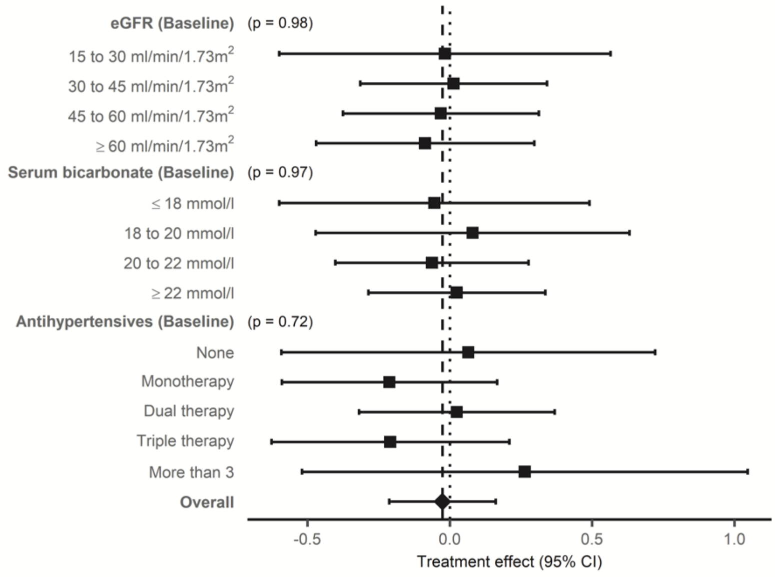

Figure S 9. Subgroup analysis of NT-proBNP. Treatment effects with 95% confidence intervals in subgroup analyses for log-transformed NT-proBNP, assessed with linear mixed models. The p-values correspond to the likelihood ratio tests comparing models with and without the interaction between treatment and subgroup. Abbreviation: eGFR, estimated glomerular filtration rate.

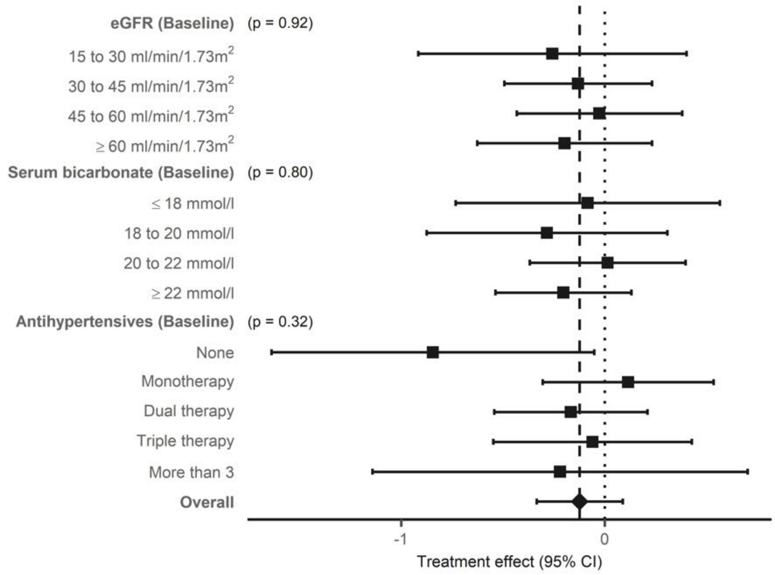

Figure S 10. Subgroup analysis of plasma renin. Treatment effects with 95% confidence intervals in subgroup analyses for log-transformed plasma renin, assessed with linear mixed models. The p-values correspond to the likelihood ratio tests comparing models with and without the interaction between treatment and subgroup. Abbreviation: eGFR, estimated glomerular filtration rate.

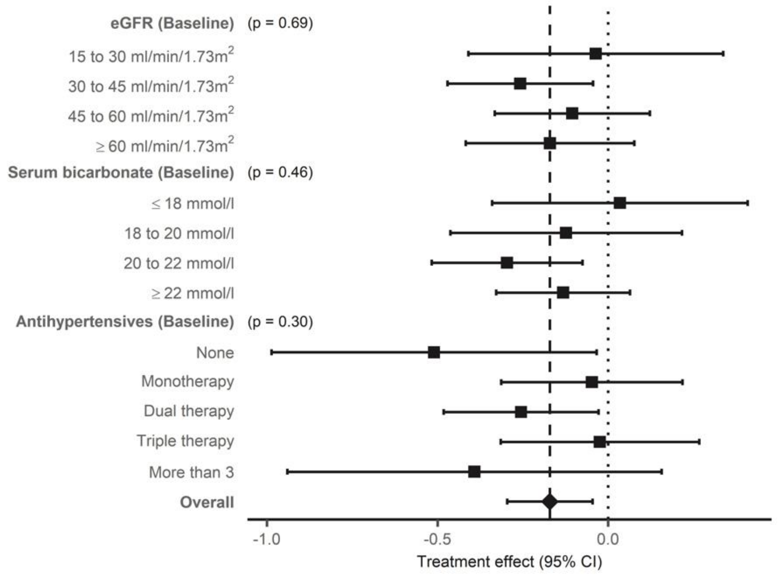

Figure S 11. Subgroup analysis of plasma aldosterone. Treatment effects with 95% confidence intervals in subgroup analyses for log-transformed plasma aldosterone, assessed with linear mixed models. The p-values correspond to the likelihood ratio tests comparing models with and without the interaction between treatment and subgroup. Abbreviation: eGFR, estimated glomerular filtration rate.

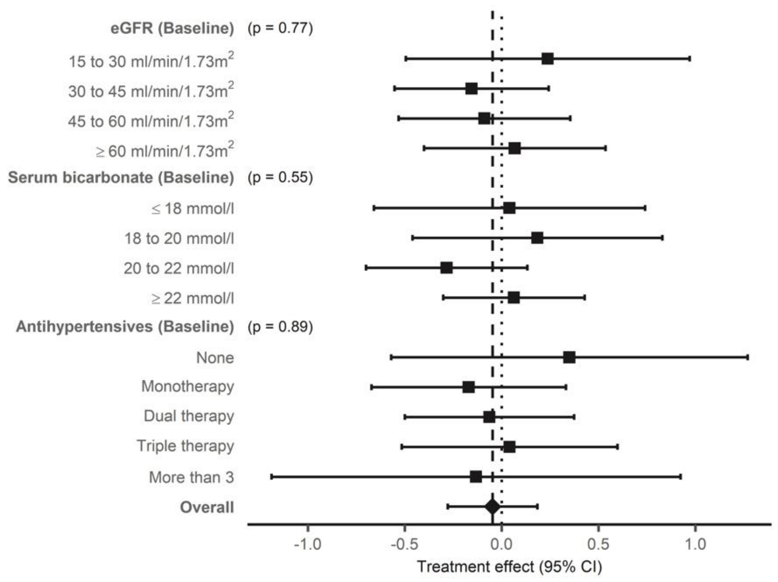

Figure S 12. Subgroup analysis of aldosterone-to-renin ratio. Treatment effects with 95% confidence intervals in subgroup analyses for log-transformed aldosterone-to-renin ratio assessed with linear mixed models. The p-values correspond to the likelihood ratio tests comparing models with and without the interaction between treatment and subgroup. Abbreviation: eGFR, estimated glomerular filtration rate.

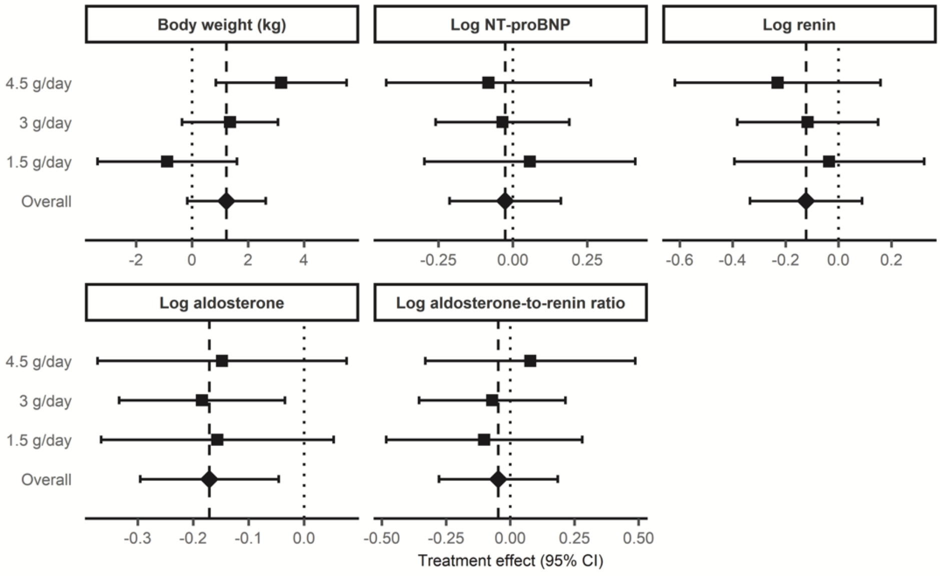

Figure S 13. Subgroup analyses of primary outcomes, stratified by dosage. Estimated effects of sodium bicarbonate dosage assigned at the dosage titration visit, compared to the placebo group, on (A) body weight (kg), (B) log-transformed NT-proBNP, (C) log-transformed plasma renin, (D) log-transformed plasma aldosterone, (E) log-transformed aldosterone-to-renin ratio. Effects are presented as differences to the placebo group with 95% confidence intervals, computed with linear mixed models.

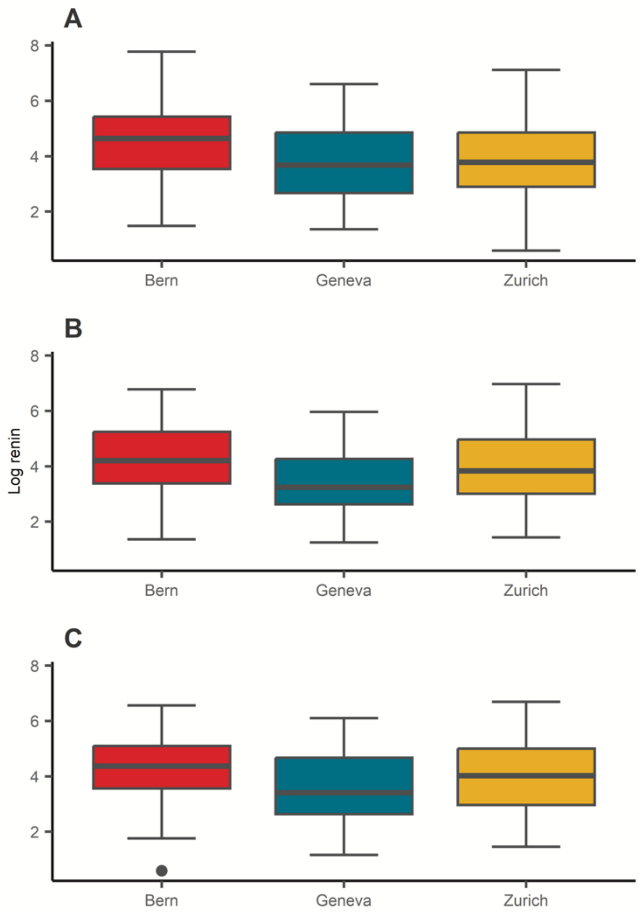

Figure S 14. Distribution of log-transformed plasma renin across 3 study centers. Log-transformed plasma renin measurements stratified by study center at (A) baseline, (B) 12 months and (C) 24 months follow-up. In Zurich, renin was immediately analyzed after blood draw. In Berne and Geneva, renin samples were frozen at -20 to -80°C before analysis.
